# A Novel PHD2/VHL-mediated Regulation of YAP1 Contributes to VEGF Expression and Angiogenesis

**DOI:** 10.1158/2767-9764.CRC-21-0084

**Published:** 2022-07-12

**Authors:** Namrata Bora-Singhal, Biswarup Saha, Durairaj Mohankumar, Jaya Padmanabhan, Domenico Coppola, Srikumar Chellappan

**Affiliations:** 1 Department of Tumor Biology, Moffitt Cancer Center, Tampa, Florida.; 2 Department of Anatomic pathology, Moffitt Cancer Center, Tampa, Florida.

## Abstract

The transcriptional coactivator YAP1 is the major oncogenic component of the Hippo signaling pathway and contributes to the genesis and progression of various tumors, including non–small cell lung cancer (NSCLC). YAP1 levels are regulated by the canonical Hippo kinases, MST1/2 and LATS1/2, which modulate its cytoplasmic retention and proteasomal degradation. While noncanonical regulation of YAP1 has been reported, its role in hypoxic response is not fully elucidated. The studies presented here show that YAP1 levels and function are modulated by von Hippel–Lindau (VHL) and prolyl hydroxylase 2 (PHD2). YAP1 could regulate multiple genes involved in angiogenesis through E2F1; it also associates with HIF1α in cancer cells under hypoxic conditions, inducing the VEGF-A promoter. Under normoxic conditions, PHD2 associates with and hydroxylates specific proline residues on YAP1, facilitating its interaction with VHL and promoting ubiquitination and subsequent proteasomal degradation. Exposure to hypoxia dissociates YAP1 from PHD2 and VHL, elevating YAP1 levels and enhancing its association with HIF1α. YAP1–HIF1α interaction was higher in NSCLC and renal cell carcinoma samples, indicating a role for this interaction in the genesis of these cancers. Our results thus reveal a novel mode of regulation of YAP1 by PHD2 and VHL in normoxic cells, suggesting that YAP1-mediated induction of VEGF and other genes contributes to hypoxic response in tumors.

**Significance:**

YAP1 under normoxic conditions is regulated by a novel nonclassical regulatory pathway involving PHD2-mediated prolylhydroxylation and proteasomal degradation; absence of this regulation under hypoxic conditions stabilizes YAP1, contributing to neoangiogenesis.

## Introduction

It is established that angiogenesis is essential for the growth of solid tumors, and environmental cues like hypoxia or nutrient deprivation can initiate angiogenesis (1–4). Hypoxia, especially, is a major driver of neoangiogenesis, which induces VEGF, primarily VEGF-A, to initiate the multi-step angiogenic process (2, 4, 5). VEGF is a diffusible mitogen that activates its receptors, VEGFR1 (Flt1), VEGFR2 (KDR/Flk1), and VEGFR3 (Flt4), which are predominantly expressed on endothelial cells, and increases vascular permeability (6–10). The key role of the VEGF receptor, KDR, in endothelial cell differentiation as well as neoangiogenesis during tumor progression, is well established (7, 10, 11). The induction of angiogenesis occurs primarily through the mediation of hypoxia-inducible factors, especially HIF1α (12). Mechanistically, HIF1α levels are downregulated in normoxic conditions by prolyl hydroxylation and subsequent degradation mediated by VHL (von Hippel–Lindau) protein; hypoxia prevents the prolyl hydroxylation of HIF1α, leading to its stabilization and the induction of VEGF (1, 13, 14).

The Hippo pathway has been implicated in the regulation of organ size as well as in cancer (15, 16). Activation of this pathway by upstream regulators Merlin/NF2 and the kinases MST1/2 and LATS1/2 inactivate the oncogenic transcriptional coactivator Yes-associated protein 1 (YAP1) or its ortholog TAZ, by retaining them in the cytoplasm and subsequent degradation (15, 17–19). This cytoplasmic sequestration and degradation occur subsequent to the phosphorylation of YAP1 at Serine 127 by LATS1/2 and is mainly mediated by the F-box-protein, βTrCP-bound SCF complex (βTrCP-SCF; refs. 20, 21). However, in the absence of these phosphorylation events, YAP1 can translocate into the nucleus and partner with different transcription factors and contribute to multiple gene expression events, resulting in cell proliferation and tumor growth. While a role for YAP1 in angiogenesis has been proposed (22–24), the underlying molecular mechanisms remain poorly understood. Here we describe a novel regulation of YAP1 under normoxic conditions, where it interacts with prolyl hydroxylase 2 (PHD2); this results in the hydroxylation of specific proline residues, leading to its interaction with the E3 ubiquitin ligase, VHL, and proteasomal degradation. On the contrary, under hypoxic conditions, the interaction with PHD2 and VHL is lost and YAP1 interacts with HIF1α and/or E2F1 to induce a variety of genes involved in the angiogenic process, including VEGF, as well as matrix metalloproteinases (MMP). YAP1–HIF1α interaction is notably higher in non–small cell lung cancer (NSCLC) tissues as compared with normal lung, suggesting a novel mechanism by which YAP1 promotes tumor growth. In addition, we find that YAP1 nuclear localization is markedly elevated in renal cell carcinoma (RCC), along with its association with HIF1α; this is significant, because VHL is often mutated and is a well-known tumor suppressor in RCC (25, 26). Taken together, our results reveal a novel noncanonical mechanism of regulation of YAP1 protein by PHD2, and the E3 ubiquitin ligase, VHL under normoxic conditions, and how YAP1 facilitates VEGF induction under hypoxic conditions.

## Materials and Methods

### Cell Lines

The human NSCLC cell lines A549 and H1650 were purchased from ATCC. A549 cells were maintained in Ham’s F12K medium (Cell Gro, Corning) supplemented with 10% FBS (VWR Life Science Seradigm) while H1650 cells were grown in RPMI1640 (Gibco, Life Technologies) containing 10% FBS. All the cultures were maintained at 5% CO_2_ at 37°C. For the hypoxia treatment, the cells were subjected to 1% O_2_ with 5% CO_2_ at 37°C for 24 hours. All the cells were routinely tested for *Mycoplasma* contamination using a PCR-based *Mycoplasma* Detection Kit (abm, #G238). Cell lines were authenticated using human short tandem repeat sequencing analyses conducted by the Molecular Genomics Core at Moffitt Cancer Center; cultured cells retrieved from the frozen stocks were discarded after every 10–15 passages.

### siRNA, Single-guide RNA, and Short hairpin RNA-mediated Depletion Assays

The two different siRNAs for YAP1 were purchased from Santa Cruz Biotechnology (sc-38637) and Thermo Fisher Scientific (107951), respectively. siRNAs For TAZ (sc-45232), MST1 (sc-39249), MST2 (sc-39247), LATS2 (sc-37444), HIF1α (sc-35561), PHD2 (sc-45537), PHD3 (sc-45799), TEAD2 (sc-45232), and TEAD4 (sc-96187) were purchased from Santa Cruz Biotechnology. siRNAs for LATS1 (137593), HIF1α (106498), TAZ (122501), TEAD2 (107179), TEAD4 (115367), PHD2 (133478), and PHD3 (128749) were purchased from Thermo Fisher Scientific. A total of 100 pmoles of siRNAs were transfected into cells using Oligofectamine reagent (Invitrogen) as per manufacturer's protocol. A nontargeting scrambled siRNA (AM4611, Ambion) was used as a control for all the transfection experiments. The cells were harvested after 48 hours after transfection for different assays. All the siRNA experiments were performed thrice. The sequence of sgRNAs against YAP1 used was as follows:

Forward primer 1: 5′ CACCGGTCGGTCTCCGAGTCCCCG 3′
Reverse primer 1: 5′ AAACCGGGGACTCGGAGACCGACC 3′
Forward primer 2: 5′ CACCGTCAGATCGTGCACGTCCGCG 3′
Reverse primer 2: 5′ AAACCGCGGACGTGCACGATCTGAC 3′

For the short hairpin RNA (shRNA)-mediated depletions, the shRNA for VHL was obtained from GE Dharmacon (RHS3979-201768476) and viruses were prepared using the psPAX2 and pMD2.G vectors in 293T cells. A549 and H1650 cells were transduced with the supernatant with viral particles and the cells were harvested after 72 hours for the indicated experiments. Additional details are provided in Supplementary Materials and Methods section.

### RNA Isolation and qRT-PCR Analysis

Total RNA was isolated from the cells by RNeasy miniprep kit from Qiagen following the manufacturer's protocol. For the sorted cells, RNeasy microprep kit from Qiagen was used. A total of 1 μg of RNA was converted into cDNA using iScript cDNA synthesis kit (Bio-Rad). The changes in the mRNA levels were analyzed using quantitative reverse-transcription PCR (qRT-PCR) that was performed using Bio-Rad CFX96 Real-time System. The primer sequences used are provided in Supplementary Materials and Methods.

### Transfections and Luciferase Assays

The A549 and H1650 cells were transiently transfected using FugeneHD (Promega) according to the manufacturer's protocol. Luciferase assays were carried out 48 hours posttransfections using the dual-luciferase assay system (Promega) according to manufacturer's instructions. Luciferase activity was measured using a luminometer (Glomax MultiJR detection system). The relative luciferase activity was measured as the ratio of the firefly luciferase to *Renilla* luciferase and the fold changes were calculated compared with the control luciferase vector alone from three independent experiments.

### Angiogenic Tubule Formation Assay

For angiogenic tubule assays in two dimensions, 100 μL of thawed Matrigel (CB-40234, Corning) was layered in the wells of 96-well TC plates followed by incubation for 30 minutes at 37°C to allow polymerization. human umbilical vein endothelial cells (HUVEC), human aortic endothelial cells (HAEC), or human microvascular endothelial cells from the lungs (HMVEC-L) were trypsinized into single-cell suspension and layered at a density 12,000 cells in 100 μL of media on polymerized matrigel in each well. Tubule formation was assessed after 6–8 hours by bright-field microscopy using an EVOS FL microscope system and images were acquired with EVOS software (Life Technologies Inc.; refs. 27–29). For studies involving siRNA transfections, 100 pmoles of each of the respective siRNAs were transfected into two 60-mm culture dish of endothelial cells (0.15 × 10^6^ cells/plate) using Oligofectamine as per manufacturer's protocol. After 48 hours of transfection, siRNA-transfected endothelial cells on one 60-mm cell culture dish were trypsinized into single-cell suspension and layered on Matrigel, under normoxic or hypoxic conditions. The tubules formed were imaged as above. The siRNA-transfected cells were analyzed for depletion efficiency by qRT-PCRs.

### Chromatin Immunoprecipitation Assays

Chromatin immunoprecipitation (ChIP) assays were conducted on asynchronous lung adenocarcinoma cell lines as described previously (30), using indicated antibodies mentioned in the Supplementary section, in detail. The interactions at the promoter were analyzed using PCR and/or q-PCR analysis. Each ChIP assay was performed twice, independently. Additional details are provided in Supplementary Materials and Methods section.

### *In Vitro* Hydroxyproline Assay

Recombinant YAP1 wild type (YAP1 WT) and YAP1 lacking residues 283–288 (∆YAP1^283–288^) or the construct with the proline residues mutated (YAP^mut^) were cloned into a GST vector and GST fusion proteins prepared; the proteins were subjected to the hydroxy proline assay using a kit (Sigma-Aldrich) as per manufacturer's protocol. The extent of proline hydroxylation was estimated from a standard curve that was generated using reagents provided in the kit.

### Proximity Ligation Assay

The asynchronously growing A549 and H1650 cells were plated on poly-D-Lysine–coated glass slides at a density of 5,000 cells per well. For the hypoxia treatment, the cells were subjected to 1% O_2_ for 24 hours and then fixed using 10% buffered formalin and permeabilized with 0.5% Triton-X-100 in PBS for 15 minutes. The cells were blocked with 5% normal goat serum in DPBS. The diluted primary antibodies [YAP1 (Abcam), HIF1α (BD Pharmingen), PHD2 (Abcam), and VHL (Cell Signaling Technology)] were added to the cells and further incubated at 4°C overnight. For the remaining steps, the Duolink assay system (Sigma-Aldrich) was used per the manufacturer's protocol (31). Detailed protocol for proximity ligation assay (PLA) experiments on tissue microarray (TMA) slides is described in the Supplementary Materials and Methods.

### Coupling of Peptides with Penetratin

A peptide corresponding to 10 residues containing the proline hydroxylation region of YAP1 (283–288 amino acids) was synthesized with a cysteine residue at the C terminus (YAP1 OH-Pro peptide: QPPPLAPQSC; Genscript). This peptide was incubated with an equimolar concentration of activated penetratin (MP Biologicals) to generate the conjugate, as per the manufacturer's protocol (32, 33). Activated penetratin was also conjugated to a scrambled peptide sequence (Scr. Peptide: QPQPLPASPC) containing the same amino acid content and was used as the control peptide for all the assays.

### IHC in TMA

IHC was conducted on a human renal cancer TMA obtained from US BioMax (BC07115a), which had 120 cores covering normal kidney (10 cores) and different grades of RCC (110 cores). Staining was performed according to previously published protocols (27, 34) using a primary YAP1 antibody (Abcam); slides were scanned on an Aperio automatic scanning system from Applied Imaging and were scored by a pathologist. The semiquantitative score was derived by considering both cellularity and intensity of expression (semiquantitative score = cellularity + intensity). Cellularity was scored as follows: a score of 3 equals to greater than 66% cellularity, a score of 2 equals to 34%–65% cellularity, and a score of 1 equals to less than 33% cellularity. Intensity was scored as follows: a score of 3 equals to strong intensity, a score of 2 equals to moderate and a score of 1 equals to weak intensity.

### Statistical Analysis

All data have been statistically analyzed using Microsoft Office Excel 2010 (Microsoft Corporation) and GraphPad Prism (GraphPad Software). The data presented here are the average ± SEM values from three independent experiments, unless otherwise mentioned. The statistical comparison between the groups was carried out by Student *t* test or two-way ANOVA with *post hoc* analysis to calculate the *P* value for statistical significance.

### Data Availability Statement

No databases were analyzed or generated in this study. The data generated in this study are available within the article and its Supplementary Data.

## Results

### YAP1 Regulates Multiple Genes Involved in Angiogenesis

**FIGURE 1:**
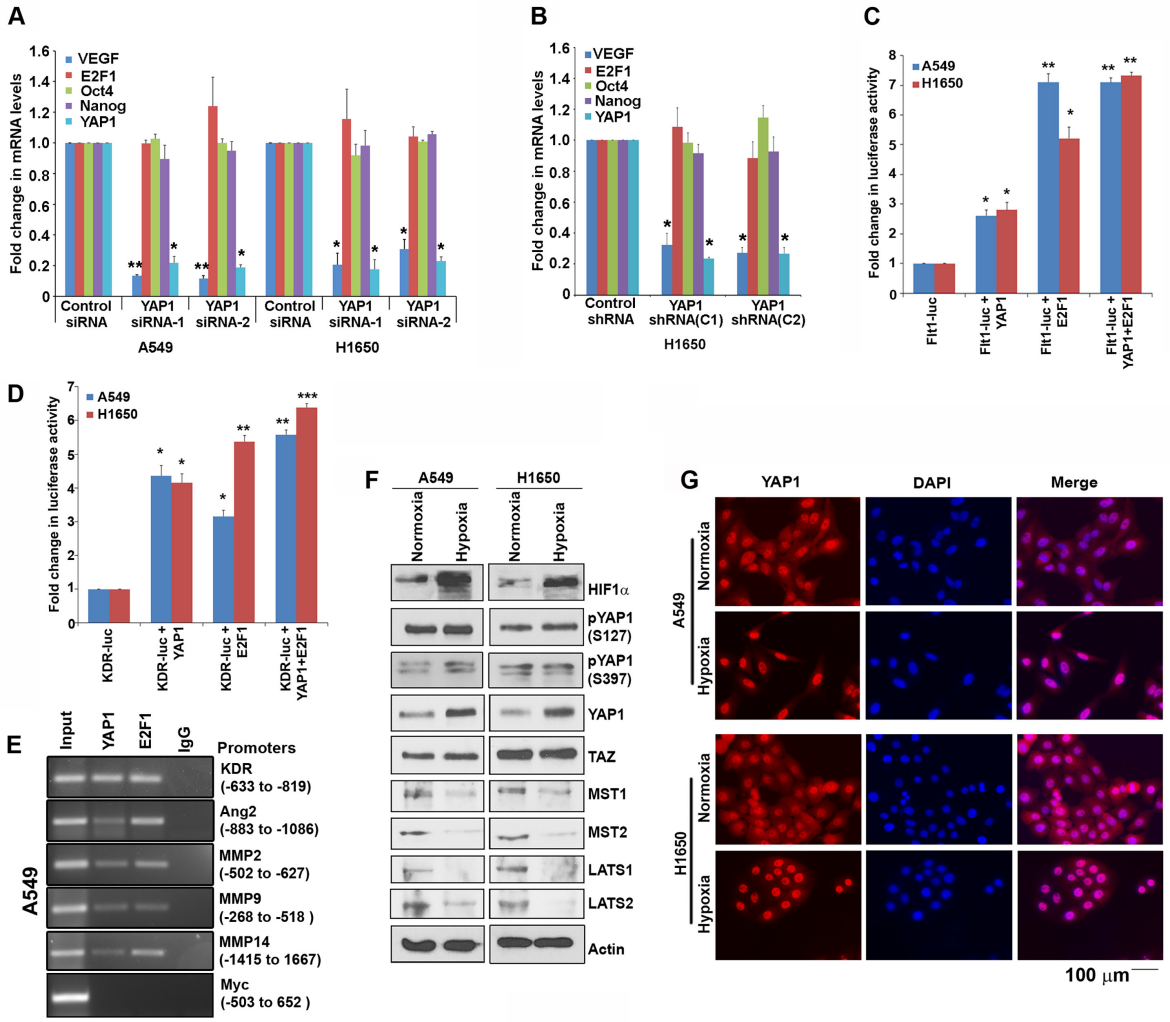
YAP1 transcriptionally regulates the expression of proangiogenic genes in NSCLC cells. **A,** Knockdown of *YAP1* using two siRNA in A549 and H1650 cells reduced the mRNA expression of *VEGF* as compared with the control siRNA-treated cells. There were no changes in *E2F1*, *Oct4* or *Nanog* mRNA expression upon *YAP1* depletion; these genes were tested as controls. **B,** Two different clones of H1650 stably expressing *YAP1*-specific shRNA showed reduction in *VEGF* mRNA expression as compared with the control shRNA-expressing cells. Expressions of *E2F1*, *Oct4,* and *Nanog* mRNA did not change upon *YAP1* knockdown. Transient transfection of Flt1 promoter-luciferase **(C)**, KDR promoter-luciferase **(D)** with YAP1 and E2F1 overexpression in both A549 and H1650 showed an additive effect on the promoter-luciferase activity. The bar graph panels represent mean ± SEM of three independent experiments. *, *P* < 0.05; **, *P* < 0.01; and ***, *P* < 0.005 derived by two-way ANOVA with *post hoc* test. **E,** ChIP assay in A549 cells with indicated antibodies showed the presence of YAP1 at E2F1 binding sites on *KDR*, *Ang2*, *MMP2*, *MMP9,* and *MMP14* promoters. *Myc* promoter was used as a negative control for the experiment. **F,** Western blot analyses on A549 and H1650 cells grown in 1% O_2_ revealed elevated expression of YAP1 as compared with normoxic cells. Although there was reduction in the levels of classical Hippo kinases, there was no significant change in phosphorylation of YAP1 at S127 and S397 sites. Also, there was no change in the expression level of TAZ in the hypoxic versus normoxic A549 and H1650 cells. HIF1α was used as a positive control. **G,** Increased nuclear localization of YAP1 in A549 and H1650 cells subjected to hypoxia (1% O_2_) for 24 hours, as detected by immunofluorescence. Scale bar, 100 μm.

Isoforms of VEGF, especially VEGF-A, promote angiogenesis primarily by activating their receptors VEGFR1 (Flt1) and/or VEGFR2 (KDR; refs. 8, 11) which are expressed on the endothelial cells; this study focused on VEGF-A. Because the expression of several genes related to oncogenesis is regulated by YAP1, we examined whether VEGF expression is modulated by YAP1 in NSCLCs. It was found that depletion of *YAP1* by two different siRNAs significantly reduced the levels of VEGF mRNA in A549 and H1650 cells, as seen by qRT-PCR (Fig. 1A). To assess whether the effect of *YAP1* knockdown was selective for the *VEGF* gene only, we conducted qRT-PCRs for other genes such as *E2F1, Oct4,* and *Nanog* (Fig. 1A; refs. 27, 35), which are not regulated by YAP1; *YAP1* depletion did not affect the expression of these genes in either A549 or H1650 cells. Similar results were obtained from H1650 cells stably transfected with two different clones of YAP*1* shRNAs; VEGF-A expression was significantly reduced in these cells as well without much reduction in E2F1, Oct4, or Nanog levels (Fig. 1B). These results indicate a regulation of *VEGF* expression by YAP1 at the transcriptional level in the NSCLC cells and this is not due to general transcriptional downregulation resulting from YAP1 depletion.

Because the depletion experiments indicated a role for YAP1 in regulating various angiogenesis-related genes, transient transfection experiments were carried out to assess whether YAP1 could induce the promoters of some of these genes. Experiments conducted on A549 and H1650 cells showed that YAP1 could induce a luciferase reporter driven by *FLT1, KDR, ANG2, MMP2, MMP9, MMP14,* and *MMP15* promoters (Fig. 1C and D; Supplementary Fig. S1A–E). It is known that VEGF receptors along with several MMPs are transcriptionally regulated by E2F1 (28, 36) and that YAP can induce genes involved in epithelial–mesenchymal transition (EMT) in lung cancer cells (27, 37). As YAP1 has also been shown to bind to E2F1 transcription factor (35, 38, 39), it was used as a positive control in these experiments. The results shown here also indicate that YAP1 might be inducing these genes through the involvement of E2F1, because transfection of YAP1 and E2F1 induced the *FLT1, KDR*, *MMP2*, *MMP9,* and *MMP14* promoters in transient transfection assays (Fig. 1C and D; Supplementary Fig. S1A–E). This was confirmed by ChIP assays on A549 cells, which detected YAP1 association on E2F-binding sites on the promoters of *KDR*, *ANG2*, *MMP2*, *MMP9,* and *MMP14* (Fig. 1E). *MYC* promoter was used as a negative control for the ChIP experiment (40). Taken together, these results suggest that YAP1 regulates the expression of multiple genes involved in angiogenesis through the mediation of E2F1 transcription factor in the tumor cells.

### Hypoxia Enhances YAP1 Levels and Promotes Its Nuclear Localization

Because YAP1 could induce VEGF and other genes involved in angiogenesis, we next investigated how hypoxia affects YAP1 levels in NSCLC cells. Western blot analyses on A549 and H1650 cells exposed to 1% oxygen for 24 hours showed that YAP1 protein levels were elevated compared with normoxic cells (Fig. 1F). Interestingly, there was no noticeable change in TAZ levels (Fig. 1F); HIF1α levels were used as a positive control. Because there was an increase in YAP1 levels, it was examined whether this correlated with a reduction in the phosphorylation of YAP1 at S127 or at S397, which are known for the cytoplasmic retention, followed by degradation of YAP1 (15, 19). Surprisingly, there was no major reduction in phospho-YAP1 at S127 or at S397 levels, indicating that the increase in the YAP1 protein levels might not be solely dependent on phosphorylation of these sites (Fig. 1F). To further examine this possibility, the proteins of the canonical Hippo pathway were investigated. There was a reduction in the protein levels of MST1/2 and LATS1/2 upon exposure to hypoxia (Fig. 1F); nevertheless, because there was no remarkable change in the phosphorylation status of YAP1, it appears that the reduction of LATS1/2 levels might not be sufficient to elevate the YAP1 protein levels. This raises the possibility that additional modulatory mechanisms beyond Hippo signaling might stabilize the YAP1 proteins, under hypoxic conditions.

Given the elevated YAP1 levels and its role in the induction of multiple angiogenic genes, experiments were conducted to assess whether exposure to hypoxia altered the subcellular localization of YAP1. Immunofluorescence experiments showed that while YAP1 protein is distributed both in the cytoplasm and the nucleus in normoxic cells, exposure to hypoxia leads to an enrichment of YAP1 in the nucleus both in the A549 and H1650 cells (Fig. 1G).

### YAP1 Is Necessary for the Formation of Angiogenic Tubules in Matrigel

**FIGURE 2:**
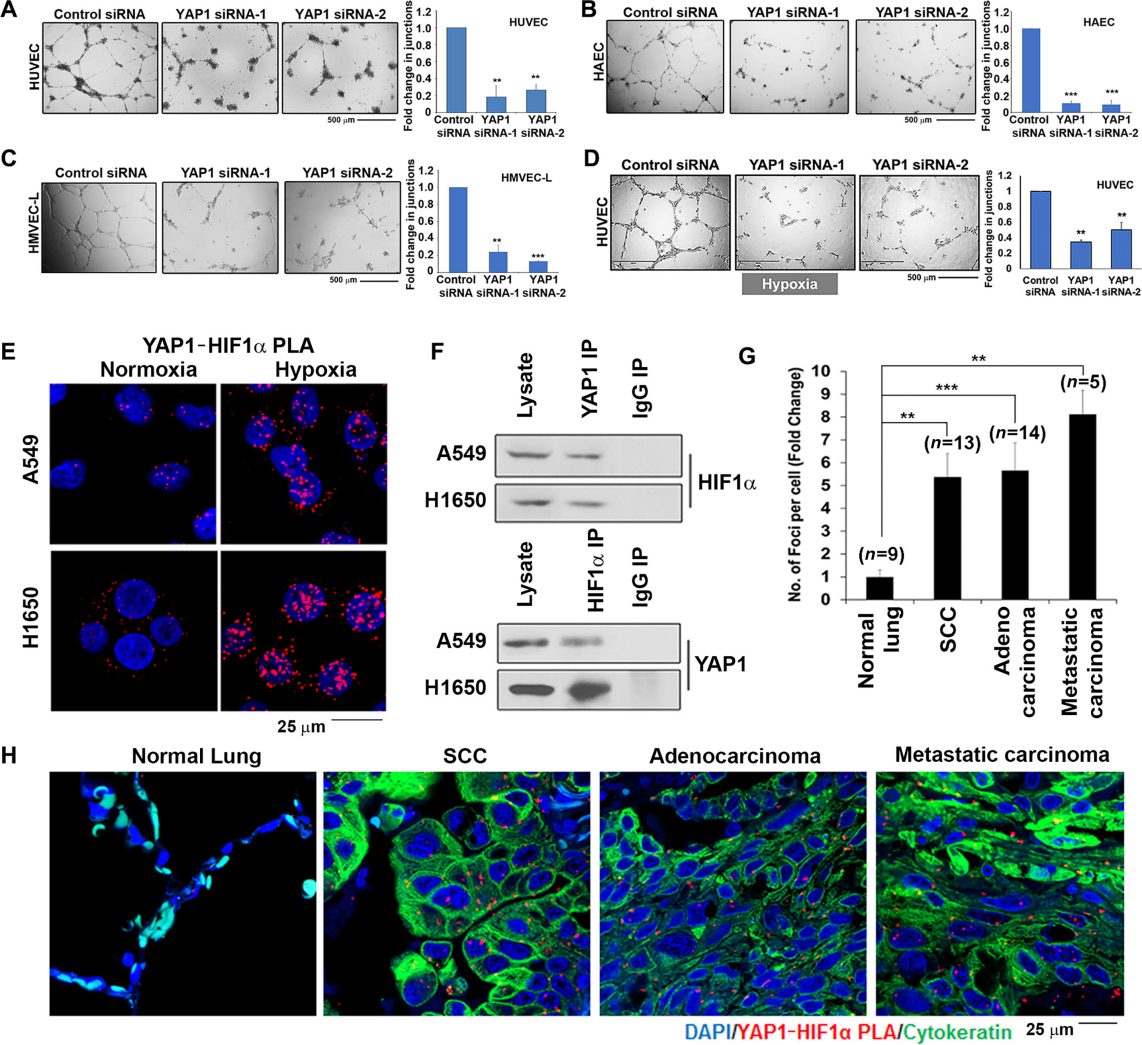
YAP1 is necessary for angiogenic tubule formation and associates with HIF1α transcription factor. **A**–**C,** Depletion of *YAP1* using two different siRNAs abrogated angiogenic tubule formation on matrigel compared with the control siRNA-transfected HUVECs, HAECs, and HMEC-Ls (scale, 500 μm). Quantification of the fold change in number of junctions formed upon *YAP1* depletion is presented graphically on the right. **D,** Depletion of YAP1 eliminated angiogenic tubule formation in HUVECs, even under hypoxic conditions. The suppression of tubule formation is quantified in the adjacent charts. **E,** A PLA showed increased interaction of YAP1 with HIF1α in A549 and H1650 cells subjected to hypoxia (1% O_2_) for 24 hours. Scale bar, 25 μm; Blue—DAPI, Red—YAP1/HIF1α PLA foci. **F,** Co-IP-Western blot assays on A549 and H1650 cells indicate that YAP1 directly associates with HIF1α protein. A reverse IP confirmed the same result with HIF1α IP and YAP1 Western blot analysis (bottom); 20% of the input was loaded in the lysate lanes. A normal IgG control IP showed minimal interaction in the co-IPs. **G** and **H,** Elevated interaction of YAP1 and HIF1α is detected in human lung tumor tissues as compared with normal lung by PLA performed on a lung TMA **(H)**. Scale bar, 25 μm; Blue—DAPI, Red—foci of YAP1/HIF1α interaction, Green - Pan-cytokeratin. The quantitation of the PLA is represented graphically **(G)**. The bar graph represents mean ± SD of the indicated number of cores, representing each tumor type. *, *P* < 0.05; **, *P* < 0.01; and ***, *P* < 0.005 derived by Student *t* test.

Because the above experiments showed a distinct role for YAP1 in the induction of several genes involved in angiogenesis, experiments were conducted to assess whether YAP1 was necessary for the formation of angiogenic tubules in matrigel. Toward this purpose, YAP1 was depleted using two different siRNAs in three different primary endothelial cells, namely HUVECs (Fig. 2A), HAEC (Fig. 2B), or HMVEC-L (Fig. 2C). Depletion of YAP1 markedly suppressed the formation of angiogenic tubules in all the three endothelial cells tested. Furthermore, depletion of YAP1 abrogated tubule formation in HUVECs, under hypoxic conditions as well (Fig. 2D). The suppression of angiogenic tubule formation was quantified by counting the number of junctions; the quantification is shown graphically on the right side of each figure panel. These results strongly suggest that the YAP1-mediated induction of genes involved in angiogenesis contributes to the formation of angiogenic tubules in matrigel.

### YAP1 Associates with HIF1α in Hypoxic Cells

The hypoxic response is mainly driven by the HIF family of transcription factors, including HIF1α (12, 41). Because depletion of YAP1 reduced VEGF levels, a PLA (31, 42, 43) was conducted to assess whether HIF1α colocalizes with YAP1 in cells. Confocal microscopy showed that YAP1 colocalizes with HIF1α under hypoxic conditions in both A549 and H1650 cells, as seen by the increased number of red foci of interactions (Fig. 2E; single antibody control is shown in Supplementary Fig. S2A); an interaction between YAP1 and HIF1α had been reported in the context of glycolysis (44). The PLA result was further supported by a double immunofluorescence experiment followed by confocal microscopy, which also showed increased colocalization of YAP1 with HIFα under hypoxic conditions in both A549 and H1650 cells (Supplementary Fig. S2B and S2C). Furthermore, immunoprecipitation (IP)-Western blot experiments conducted on A549 and H1650 cells subjected to hypoxia showed that YAP1 could be detected in HIF1α immunoprecipitate and *vice versa* (Fig. 2F). There was no detectable protein in IP conducted with a control IgG, confirming that YAP1 physically interacts with HIF1α in both A549 and H1650 cells. These results indicate that YAP1 is not only upregulated, but also associated with HIF1α under hypoxic conditions.

We next investigated the association of YAP1 with HIF1α in lung tumor tissues by performing a PLA on a lung tumor TMA. The results showed that there was a significantly higher interaction of YAP1 and HIF1α in lung adenocarcinomas as well as lung squamous cell carcinomas compared with normal tissues (Fig. 2G and H). The interaction could also be detected in adenocarcinomas that metastasized to bone and lymph nodes (Fig. 2G and H; combined data from both the metastatic sites). This interaction could be detected in surrounding stromal cells as well, suggesting that this association might be reflective of a hypoxic tumor microenvironment.

### YAP1 Regulates VEGF Gene Expression in Association with HIF1α

**FIGURE 3:**
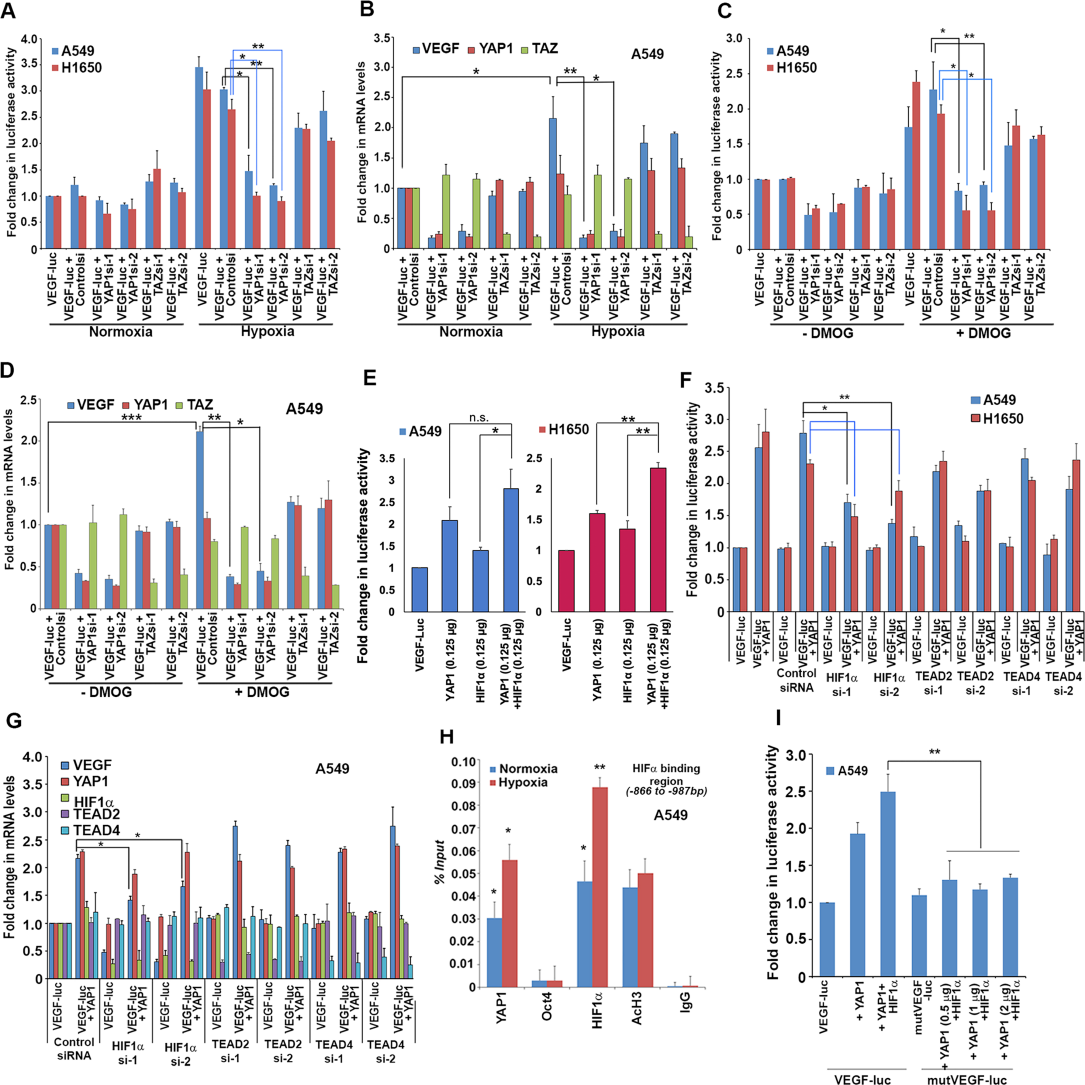
YAP1 regulates *VEGF* expression in association with HIF1α. **A,** Depletion of *YAP1,* but not *TAZ,* resulted in decreased *VEGF* promoter luciferase activity in hypoxic A549 and H1650 cells in a transient transfection assay. **B,** qRT-PCRs showed decreased expression of endogenous *VEGF* mRNA in A549 cells transfected with *YAP1* siRNA and subjected to hypoxia. Such a change was not observed when *TAZ* was depleted in the A549 cells. **C,** Similarly, depletion of *YAP1,* but not *TAZ,* decreased *VEGF* promoter luciferase activity in A549 and H1650 cells treated with 1 mmol/L DMOG as compared with control vehicle-treated cells. **D,** The expression of endogenous *VEGF* mRNA was reduced when A549 cells were transfected with *YAP1* siRNA but not *TAZ* siRNA; cells treated with 1 mmol/L DMOG showed the same result. **E,** YAP1 and HIF1α overexpression showed an increased effect on *VEGF* promoter luciferase activity in transient transfections in A549 and H1650 cells. **F,** Loss of *VEGF-luc* promoter luciferase activity in transient transfection assays upon *HIF1α* depletion when YAP1 was overexpressed; no significant change was observed with the depletion of *TEAD2* or *TEAD4* using two different siRNAs for each. **G,** qRT-PCRs showed an increase in endogenous *VEGF* mRNA expression in A549 cells when YAP1 was overexpressed*.* However, this increase in endogenous *VEGF* mRNA was less when *HIF1α* was depleted in the presence of YAP1. The depletion of *TEAD2* or *TEAD4* did not affect the expression of endogenous *VEGF* mRNA expression, when YAP1 was overexpressed. **H,** ChIP-qPCR analysis performed in normoxic and hypoxic A549 cells with the indicated antibodies showed increased presence of YAP1 at the HIF1α binding sites on *VEGF* promoter at position −866 to −987 bp, upstream of transcription start site in the hypoxic A549 cells. **I,** A site-directed mutation in the HIF1α binding site on the *VEGF* promoter (mutVEGF-Luc) abrogated promoter luciferase activity induced by YAP1. The bar graphs in this panel represent mean ± SEM of three independent experiments. *, *P* < 0.05 and **, *P* < 0.01 derived by two-way ANOVA with *post hoc* test.

Earlier reports had suggested that YAP1 has a role in regulation of VEGF-mediated angiogenesis during development (23). Because our experiments indicate that YAP1 regulates multiple angiogenic genes and is also upregulated during hypoxia, experiments were conducted to assess if YAP1 contributes to VEGF expression in hypoxic lung cancer cells. In the first set of experiments, A549 and H1650 cells were transiently transfected with a *VEGF* promoter-luciferase reporter (VEGF-luc), along with a control siRNA or two different siRNAs against *YAP1*. Subjecting the cells to hypoxic conditions led to an increase in luciferase activity; however, this increase was lost in both A549 and H1650 cells when *YAP1* was depleted (Fig. 3A). Interestingly, such a reduction in VEGF-luc activity was not observed when *TAZ* was depleted in either cell line (Fig. 3A). The mRNA expression of endogenous *VEGF* was evaluated in these siRNA-transfected A549 and H1650 cells. The results showed a decrease in endogenous *VEGF* mRNA expression upon *YAP1* depletion, but not with *TAZ* depletion, in A549 (Fig. 3B) and H1650 cells (Supplementary Fig. S3A) in normoxia as well as hypoxia. The knockdown efficiency of the *YAP1* siRNAs and *TAZ* siRNAs was also confirmed by qRT-PCRs in A549 and H1650 cells (Fig. 3B; Supplementary Fig. S3A). Treatment of A549 and H1650 cells, transiently transfected with a VEGF-luc reporter with 1 mmol/L of dimethyloxalylglycine (DMOG), a PHD2 inhibitor, also induced the VEGF-luc reporter, whereas depletion of YAP1 abolished the induction of the VEGF promoter (Fig. 3C; Supplementary Fig. S3B). Similarly, depletion of YAP1, but not TAZ, significantly reduced the ability of DMOG to induce endogenous VEGF mRNA expression, as seen by a qRT-PCR analysis (Fig. 3D). These results suggest that YAP1 has a unique function in the induction of VEGF promoter.

Because HIF1α was found to associate with YAP1 in hypoxic conditions, we investigated whether YAP1 co-operated with HIF1α to induce the *VEGF* promoter. Transient transfection experiments showed that YAP1 co-operated with HIF1α to elevate *VEGF* promoter-luciferase activity in both A549 and H1650 cells (Fig. 3E). YAP1 is a known transcriptional coactivator and has been reported to act through TEAD family members of transcription factors, especially TEAD2 and TEAD4 (45–48). Transient transfections were conducted to assess whether YAP1 induced VEGF-luc through its interaction with HIF1α or through TEAD2 or TEAD4, by depleting either *HIF1α*, *TEAD2,* or *TEAD4* using two different siRNAs. The results showed that while overexpression of YAP1 induced the promoter activity of VEGF-luc, the depletion of *HIF1α* reduced this induction significantly in both A549 and H1650 cells (Fig. 3F). However, no such reduction was observed when *TEAD2* or *TEAD4* were depleted (Fig. 3F), suggesting that YAP1 induces the VEGF promoter activity predominantly through HIF1α. The depletion of *HIF1α*, *TEAD2,* and *TEAD4* upon siRNAs treatment was also confirmed by qRT-PCRs in both the cell lines (Fig. 3G; Supplementary Fig. S3C).

An analysis of *VEGF* promoter using the Alggen online promoter analysis tool (http://alggen.lsi.upc.es/) identified the binding sites for HIF1α on the *VEGF* promoter which had been reported previously (49). ChIP analysis was carried out to confirm the binding of YAP1 to the VEGF promoter at the HIF1α binding site; ChIP-qPCR analysis showed the presence of both YAP1 and HIF1α on the same region of the *VEGF* promoter (Fig. 3H), though there is an unlikely possibility that the recruitment occurs outside the HIF1α binding site. Acetylated histone H3 was used as a positive control and nonspecific IgG was used as a negative control for these experiments (Fig. 3H). Next, we mutated the HIF1α binding site on the *VEGF* promoter-luciferase reporter construct (mutVEGF-luc) and carried out similar transient transfection assays. Cotransfecting YAP1 and HIF1α showed a dosage-dependent increase in the activity of wild-type VEGF-luc; in contrast, there was no induction of mutVEGF-luc promoter, even in the presence of increasing concentrations of YAP1 (Fig. 3I). These observations indicate that YAP1 can act as a transcriptional coactivator of HIF1α to regulate the expression of VEGF.

### Novel Noncanonical Regulation of YAP1 by Prolyl Hydroxylase 2

**FIGURE 4:**
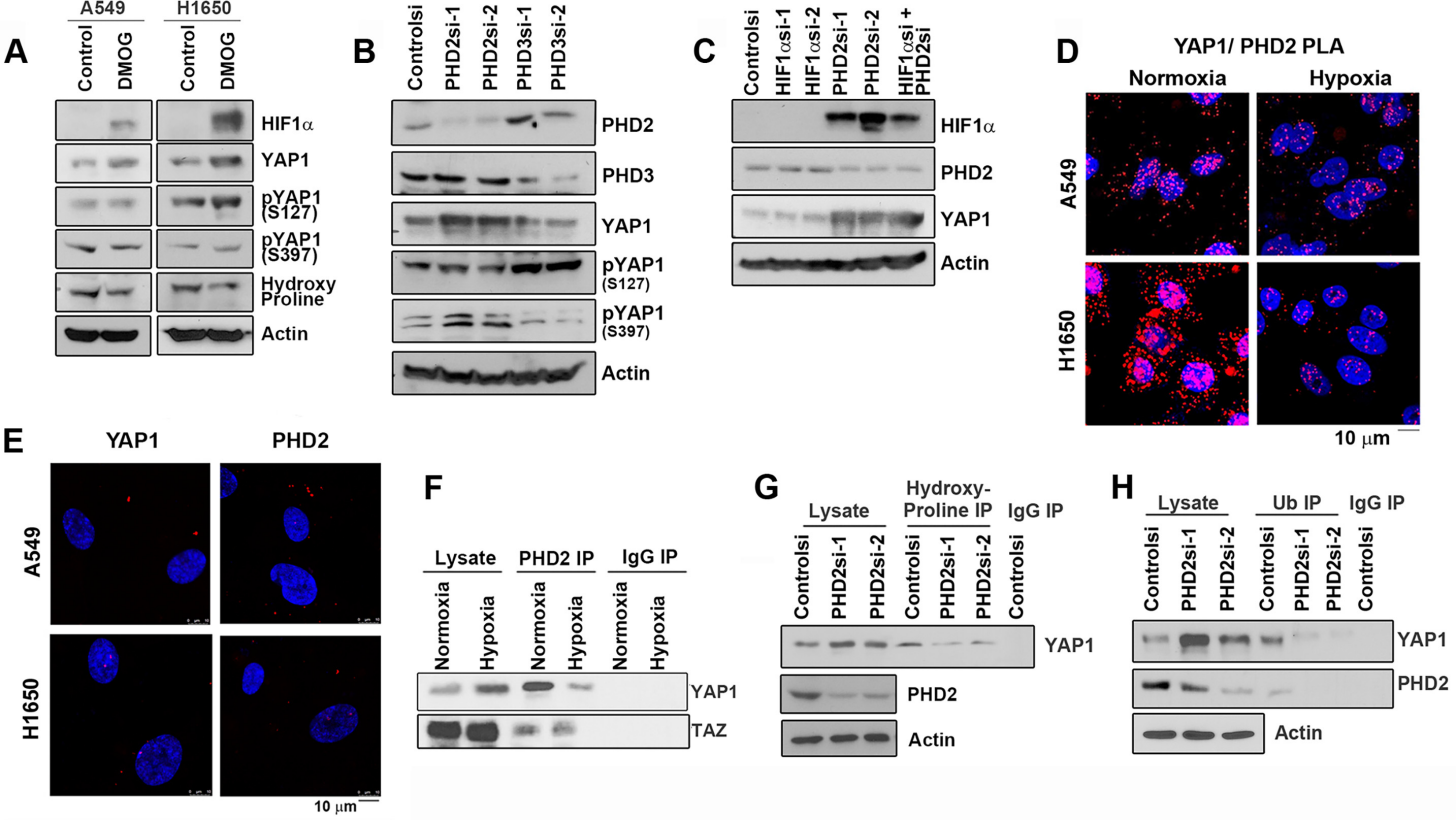
PHD2 mediates proline hydroxylation of YAP1. **A,** Treatment with 1 mmol/L DMOG for 24 hours elevated YAP1 protein levels in A549 and H1650 cells as compared with control vehicle treated cells, as seen by Western blotting. There was no notable change in YAP1 phosphorylation. **B,** Depletion of *PHD2,* but not *PHD3,* with two different siRNAs in A549 cells showed an increase in YAP1 protein levels as compared with control siRNA-transfected cells. **C,** Depletion of *PHD2*, but not *HIF1α,* elevates the levels of total YAP1 in A549 cells. Depleting *HIF1α* along with *PHD2* also elevated YAP1 levels under normoxic conditions. **D,** PLA revealed that YAP1 associates with PHD2 and this interaction decreases upon hypoxia (1% O_2_) exposure for 24 hours in both A549 and H1650 cells. Scale bar, 10 μm, Blue—DAPI, Red—YAP1/PHD2 PLA foci. **E,** Single YAP1 or PHD2 antibody used as negative controls in the above PLA experiment. **F,** Co-IP assay in normoxic and hypoxic cells further confirmed that YAP1 directly interacts with PHD2 and the association is decreased in hypoxic cells. TAZ was also found to associate with PHD2 but no significant difference was observed in its levels between normoxic and hypoxic conditions. **G,** Depletion of *PHD2* by two different siRNAs eliminates the prolyl hydroxylation of YAP1, as seen in an IP-Western blot experiment on A549 cells. **H,** A ubiquitin IP in *PHD2*-depleted A549 cells showed a decrease in ubiquitination of YAP1 proteins compared with the control siRNA treated cells.

The expression and localization of YAP1 is primarily regulated by the Hippo signaling pathway through a series of phosphorylation events in most developmental paradigms (17, 20, 50). Because there was no reduction in the levels of YAP1 phosphorylation concomitant with the increase in its protein level during hypoxia, we investigated additional noncanonical mechanisms that might be involved in the YAP1 upregulation. We first investigated whether the prolyl hydroxylase inhibitor, DMOG (51) could upregulate YAP1 protein levels. Western blotting on A549 and H1650 cells treated with 1 mmol/L DMOG for 24 hours showed that the treatment increased the levels of both YAP1 as well as HIF1α (Fig. 4A). Hence, we next examined whether depletion of the prolyl hydroxylase enzymes, PHD2 as well as PHD3, which regulate HIF1α in response to oxygen levels (52–55), would result in YAP1 upregulation under normoxic conditions. A Western blot analysis showed that depletion of PHD2 by two different siRNAs upregulated YAP1 protein in A549 cells; there was no notable change in the levels of YAP1 when PHD3 was depleted (Fig. 4B). To distinguish the role of PHD2 and HIF1α in upregulation of YAP1, we carried out a depletion experiment with *PHD2* siRNA, *HIF1*α siRNA or both in combination. The results clearly showed that YAP1 levels were elevated in the absence of PHD2 even when HIF1α expression was abrogated (Fig. 4C). In addition, the depletion of HIF1α alone did not change the expression of YAP1 (Fig. 4C).

Because PHD2 depletion affects YAP1 levels, we assessed whether they associate with each other. A double immunofluorescence experiment showed that YAP1 colocalized with PHD2 in normoxia; the colocalization was reduced under hypoxic conditions (Supplementary Fig. S4A)**.** PLA experiments confirmed that YAP1 and PHD2 protein interact with each other under normoxic conditions (Fig. 4D); Fig. 4E shows the single antibody control, where only negligible signal was observed. Finally, co-IP experiments were carried out on A549 cells to confirm this interaction. There was a notable association of YAP1 with PHD2 under normoxic conditions, while the interaction was decreased under hypoxia (Fig. 4F), or upon treatment with visudyne (verteporfin), the known YAP1 inhibitor (47); visudyne reduced the levels of both the proteins as well (Supplementary Fig. S4B). Interestingly, a minimal interaction of TAZ with PHD2 was also detected in normoxic cells; however, there was no marked increase in the TAZ levels in hypoxic cells (Fig. 4F). To support the hypothesis that YAP1–HIF1α and YAP1–PHD2 interactions do exist *in vivo*, we have utilized mouse lung-xenograft tissues. PLA data in Supplementary Fig. S5A and S5B clearly show abundant YAP1-HIF1α association present in the lung tumor xenograft tissues compared with YAP1–PHD2 interaction; this suggests that a HIF1α-YAP1 functional network is active within the tumors, *in vivo*.

Given the physical association of PHD2 with YAP1, we examined whether proline residues are hydroxylated on YAP1. Interestingly, proline hydroxylation on YAP1 protein could be detected by Western blot analysis using YAP1 antibody on an IP experiment conducted with an anti-hydroxyproline antibody, suggesting that YAP1 indeed is hydroxylated on proline residues (Fig. 4G). Furthermore, we find that depletion of PHD2 by two different siRNAs eliminated the prolyl hydroxylation of YAP1, as seen by the same IP-Western blot analysis (Fig. 4G), strongly indicating that PHD2 mediates prolyl hydroxylation of YAP1 under normoxic conditions.

Because PHD2 is known to regulate HIF1α and target it for ubiquitination and degradation (13, 26), IP-Western blot experiments were conducted to assess whether depletion of PHD2 affected the ubiquitination of YAP1. It was found that depletion of *PHD2* by two different siRNAs markedly reduced the ubiquitination of YAP1 in normoxic cells; there was concomitant increase in the levels of YAP1, as seen in the input lanes (Fig. 4H). This confirms that binding of PHD2 to YAP1 leads to hydroxylation of prolines, resulting in YAP1 ubiquitination and degradation.

### YAP1 and PHD2 Interaction may Regulate Angiogenesis

**FIGURE 5:**
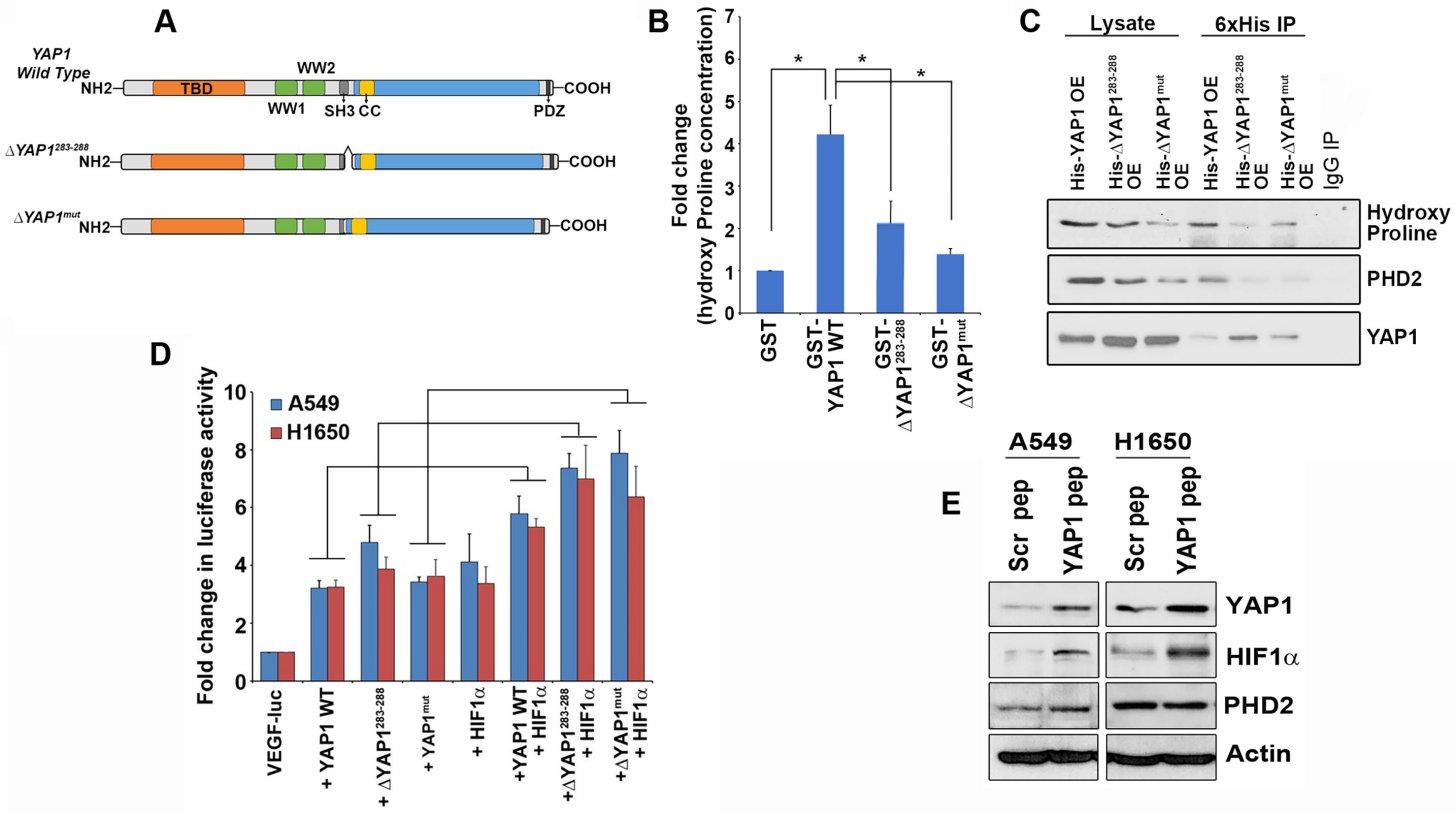
Proline hydroxylation of YAP1 regulates angiogenic functions. **A,** Schematic to represent YAP1 domains (YAP1 WT), YAP1 mutant with deletion of aa 283 to aa 288 to eliminate the proline hydroxylation (∆YAP1^283–288^) sites and a YAP1 with point mutations in the region aa 283 to aa 288 (∆YAP1^mut^). **B,** An *in vitro* proline hydroxylation assay detected a decrease in proline hydroxylation of GST-∆YAP1^283–288^ and GST-∆YAP1^mut^ as compared with GST-YAP1 WT. The bar graph represents mean ± SEM of three independent experiments. *, *P* < 0.05 derived by Student *t* test. **C,** An overexpression of His-tagged YAP1 WT, His-tagged ∆YAP1^283–288^, and His-tagged ∆YAP1^mut^ followed by IP with an anti-6XHis antibody showed a decrease in the proline hydroxylation of both ∆YAP1^283–288^ and ∆YAP1^mut^ mutant. **D,** Increase in VEGF promoter luciferase activity with the ∆YAP1^283–288^ and ∆YAP1^mut^ as compared with the YAP1 WT when cotransfected with HIF1α. The bar graphs in this panel represent mean ± SEM of three independent experiments. *, *P* < 0.05 derived by two-way ANOVA with *post hoc* test. **E,** Western blot analyses of A549 and H1650 cells treated with a peptide against YAP1 proline hydroxylation site (YAP1 OH-Pro peptide) conjugated to a carrier peptide, penetratin showed increase in YAP1 protein levels.

An analysis of the YAP1 protein sequence for hydroxylation revealed a site from aa 283 to aa 288 that has the potential to be hydroxylated (52, 53). On the basis of this observation, YAP1 constructs were generated with deletion of residues, aa283–288 (∆YAP1^283–288^) and with point mutations of the site (YAP1^mut^; Fig. 5A). An *in vitro* hydroxylation reaction using recombinant WT YAP1, with YAP1 protein lacking residues aa283–288 (∆YAP1^283–288^), or with point mutation of the putative hydroxylation site (YAP1^mut^) showed that the levels of proline hydroxylation were significantly reduced when ∆YAP1^283–288^ or YAP1^mut^ was used compared to the WT YAP1, suggesting that this region indeed is targeted for proline hydroxylation (Fig. 5B). Unconjugated GST was used as a control in the experiment (Fig. 5B). Subsequently, His-tagged WT-YAP1, ∆YAP1^283–288^, and YAP1^mut^ were overexpressed in asynchronous A549 cells and an IP was carried out with an anti-His antibody. Western blot analysis showed that the levels of prolyl hydroxylation was reduced when residues 283–288 were deleted or mutated, suggesting that this region is one of the major sites targeted by PHD2 (Fig. 5C). The functional consequence of deleting or mutating these residues was examined in a transient transfection experiment. The results showed an increase in VEGF-luc promoter activity in the presence of WT YAP1, while ∆YAP1^283–288^ showed a minimal increase in the VEGF promoter activity (Fig. 5D). The cotransfection of HIF1α along with YAP1 WT, ∆YAP1^283–288^ and YAP1^mut^ also showed an increase in the VEGF promoter activity (Fig. 5D).

To further confirm the functional significance of the interaction of PHD2 with YAP1 and the relevance of the region, spanning aa283–288, cells were treated with a peptide corresponding to the YAP-hydroxylation domain conjugated to the carrier peptide, penetratin (27, 32, 33), to disrupt the binding of PHD2 to the endogenous YAP1. Western blot analyses in A549 and H1650 cells treated with the peptide-penetratin conjugate showed an increase in YAP1 protein levels, compared with cells treated with a control peptide conjugate (Fig. 5E). Interestingly, there was also an increase in the levels of HIF1α (Fig. 5E). Together, these results suggest that YAP1 is hydroxylated by PHD2 at region aa283–288, leading to its degradation in normoxic cells; in contrast, this interaction is reduced under hypoxic conditions, leading to an elevation of YAP1 levels and enhancing its co-operation with HIF1α to promote angiogenesis.

### YAP1 Interacts with the E3 Ubiquitin Ligase, VHL

**FIGURE 6:**
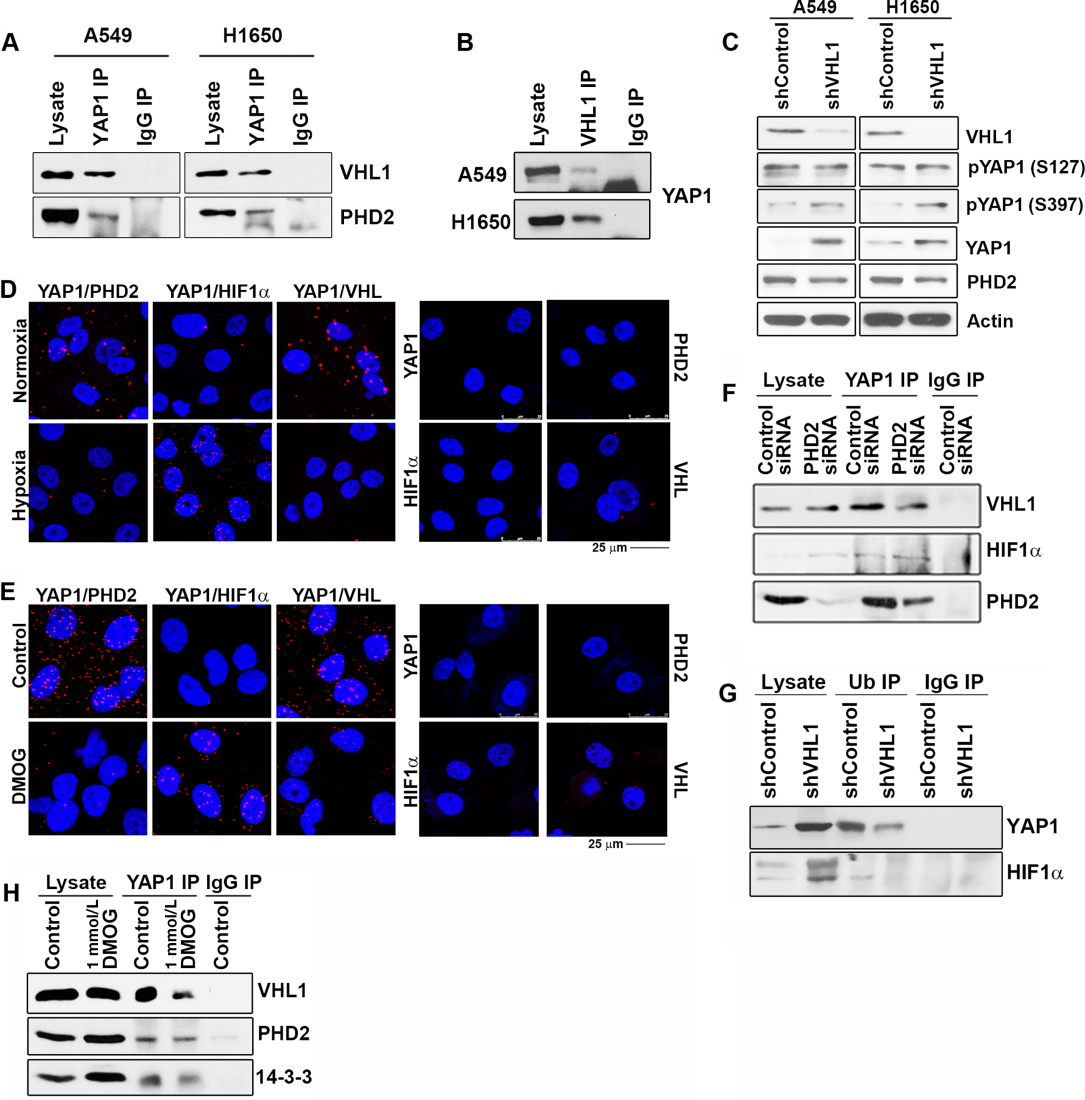
YAP1 associates with VHL E3 ligase. **A,** Co-IP Western blot experiments showed a direct association of YAP1 with VHL protein in both A549 and H1650 cells. **B,** A reverse IP confirmed the interaction of YAP1 with VHL. **C,** Depletion of *VHL* with shRNA in A549 and H1650 cells elevated YAP1 protein levels. There was marginal increase in the phosphorylation of YAP1 at pYAP1 S397 site. No change was observed in the expression of PHD2. PLA performed on A549 cells showed a decrease in the association of YAP1 with VHL during hypoxia (**D**) or in DMOG treatment (**E**). Corresponding increase in the interaction of YAP1 with HIF1α during hypoxia was observed. The right panels represent the single antibody controls for hypoxia (**D**) and DMOG (**E**) treatment. Scale bar, 25 μm. **F,** IP assays showed decreased association of YAP1 with VHL in the absence of PHD2. HIF1α Western blot analysis showed no change in the interaction of YAP1 with HIF1α upon *PHD2* depletion. **G,** IP-Western blot analysis with a ubiquitin antibody in *VHL*-depleted cells showed a decrease in YAP1 ubiquitination as compared with shControl transfected cells. The HIF1a Western blot analysis was used as a positive control in this experiment. **H,** IP assays also showed decreased interaction of YAP1 with VHL in DMOG-treated A549 cells. There was also decrease in YAP1 association with PHD2 and 14-3-3 protein in the DMOG-treated cells.

Role of the tumor suppressor, VHL protein in downregulating HIF1α levels under normoxic conditions is well established (13, 14, 25, 56). VHL, a E3 ubiquitin ligase, targets HIF1α for degradation under normoxia upon hydroxylation at its proline residues by PHD2. Given that YAP1 is upregulated similar to HIF1α under hypoxia and interacts with PHD2 under normoxia, we investigated whether YAP1 can interact with VHL. IP-Western blot analysis carried out on lysates from A549 and H1650 cells detected an interaction of YAP1 with VHL in both the cell lines (Fig. 6A); an IP-Western blot experiment in the reverse direction, using an anti-VHL antibody for IP, and probing for YAP1 further confirmed the result (Fig. 6B). Furthermore, depletion of *VHL* in A549 and H1650 cells using shRNA led to an elevation of YAP1 levels (Fig. 6C). The interaction of YAP1 with VHL was confirmed using PLAs in cells grown under normoxic and hypoxic conditions. While there was a substantial interaction of YAP1 with VHL under normoxic conditions, this was reduced under hypoxic conditions; similar results were observed in the YAP1–PHD2 interaction (Fig. 6D). Conversely, the interaction of YAP1 with HIF1α was lower in the normoxic cells but elevated under hypoxic conditions, as expected (Fig. 6D). Similar changes in interactions between YAP1 with either VHL or PHD2 were also observed when cells were treated with the prolyl hydroxylase inhibitor, DMOG (Fig. 6E). Here, the interaction of YAP1 with VHL or PHD2 decreased significantly upon DMOG treatment with a concomitant increase in its association with HIF1α (Fig. 6E). Single antibody treatments were used as negative control for the PLA (Fig. 6D and E, right). Next, it was examined whether PHD2 was necessary for YAP1 to interact with VHL. Toward this purpose, *PHD2* was depleted by siRNA transfection and an IP-Western blot experiment was conducted to assess the association of YAP1 with VHL (Fig. 6F). There was a decrease in the interaction of YAP1 with VHL in the absence of PHD2; at the same time, there were no significant changes in YAP1 interaction with HIF1α when *PHD2* was depleted (Fig. 6F). We also carried out an IP-Western blot experiment to assess ubiquitination of YAP1 in *VHL*-depleted cells; there was decreased ubiquitination of YAP1 in the absence of VHL protein (Fig. 6G). The ubiquitination status of HIF1α was also checked as a control; this was also reduced, as expected (Fig. 6G). Similarly, an IP-Western blot experiment showed a decrease in interaction of YAP1 with VHL and PHD2 in DMOG-treated cells (Fig. 6H); YAP1 interaction with 14-3-3 protein was also marginally decreased (Fig. 6H). Taken together, these data clearly indicate that YAP1 can be regulated by a novel noncanonical mechanism involving proline hydroxylation of YAP1 by PHD2, which targets YAP1 for degradation by promoting its interaction with the E3 ubiquitin ligase, VHL.

### Nuclear Localization of YAP1 and Increased YAP1–HIF1α Interaction in Human RCC Tissues

**FIGURE 7:**
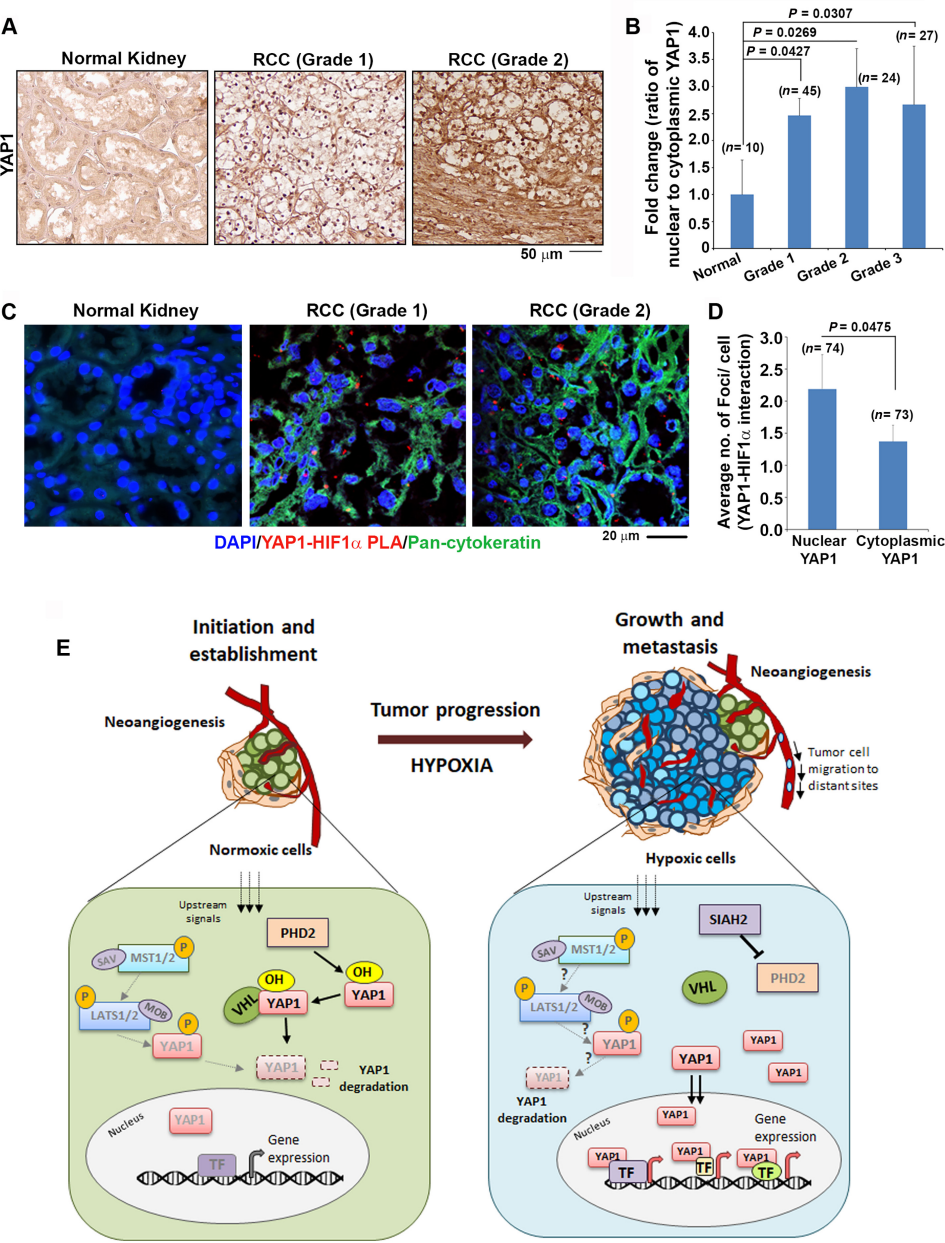
YAP1 nuclear localization in RCC. **A** and **B,** IHC analyses of a tumor microarray showed an increased nuclear YAP1 in human RCC tissues as compared with normal kidney (**A**) and its quantitation is represented graphically (**B**). Scale bar, 50 μm. The bar graph represents mean ± SD of the indicated number of cores representing the grade of tumor. *P* values were derived by Student *t* test. **C,** Elevated interaction of YAP1 with HIF1α observed in the human RCC tumor tissue samples as detected by PLA. Scale bar, 20 μm, Blue –DAPI, Red – YAP1/HIF1α PLA, Green- Pan-cytokeratin. **D,** Quantitation of the YAP1-HIF1α interaction in the RCC tumor tissues showed increased interaction in the nucleus in the tumor areas; there was relatively less cytoplasmic YAP1 in the tumor tissues. The bar graph represents mean ± SD in localization of YAP1 (nuclear and cytoplasmic) for the indicated number of cores. *P* values were derived by Student *t* test. **E,** Schematic to represent the proposed model of YAP1 regulation in normoxia versus hypoxia during tumor progression and its possible role in tumor angiogenesis.

It is established that loss of the tumor suppressor protein, VHL plays a crucial role in RCC (25, 26, 57). As a proof of principle, we next investigated the status of YAP1 in RCC. An IHC analysis of YAP1 on a TMA of RCC samples showed an increase in YAP1 nuclear localization in RCC tissues compared with normal kidney (Fig. 7A and B). Furthermore, moderate and poorly differentiated tumors had more nuclear YAP1 as compared with the well-differentiated tumors; this was confirmed by the quantitation of the nuclear to cytoplasmic ratio of YAP1 in different grades of RCC (Fig. 7B). We also observed YAP1 staining in the tumor stroma. Next, a PLA was conducted to assess the interaction of YAP1 with HIF1α in the RCC tissue samples. There was a marked increase in the interaction of YAP1 with HIF1α in the tumor samples compared with normal tissues (Fig. 7C). Quantitation of YAP1 interaction with HIF1α (red foci) clearly showed an increase in this interaction in RCC tumor tissues, where distribution of YAP1 was more nuclear than cytoplasmic (Fig. 7D). Taken together, the data presented here show that YAP1 can be regulated by a novel nonclassical pathway under normoxic conditions, which involves PHD2 and VHL; absence of this regulation under hypoxic conditions stabilizes YAP1 protein and contributes to angiogenesis and cancer progression.

## Discussion

Hippo signaling pathway is regulated by diverse upstream stimuli such as G protein–coupled receptors, cell polarity and density, mechanical stress, growth factors, and cellular energy stress (15, 17, 50, 58). Hippo pathway components are indispensable during development, whereas, the Hippo pathway effector protein, YAP1, contributes to the genesis and progression of various cancers (18, 59–62). At the same time, the regulatory role of YAP1 in angiogenesis and how it is regulated under hypoxic versus normoxic conditions remain relatively unknown. Our study highlights a novel regulation of YAP1 by a previously unidentified noncanonical mechanism involving proline hydroxylation and VHL-dependent proteasomal degradation.

The developing tumor presents a challenge in supplying nutrients and oxygen to the growing mass of the fast-dividing cells. As a result of the high metabolic demands, the tumor microenvironment is known to be highly hypoxic in nature; hypoxia is a well-known trigger for the process of neoangiogenesis (2, 5). Our results clearly show an increase in YAP1 protein levels as well as its nuclear localization upon exposure to hypoxia. Interestingly, these observations are distinct from an earlier report which has shown a decrease in LATS2 kinase activity leading to stabilization of YAP1 under hypoxia, by a reduction in pYAP1 S127 phosphorylation (63). Contrary to this report, we did not observe any reduction in the phosphorylation status of YAP1 although there was a decrease in both LATS1 and LATS2 levels; these differences could be due to the cell types used. In addition, it should be mentioned that there is a basal level of YAP1 in normoxic cells, which essentially modulates other downstream events regulated by the classic Hippo pathway. It is likely that this basal YAP1 responds to cell density and mechanotransduction, as has been demonstrated in several systems (64, 65); in addition, YAP1 has been reported to alter certain metabolic pathways in cells (66). At the same time, YAP1 upregulation upon exposure to hypoxia elevates its levels, facilitating its interaction with HIF1α to modulate genes involved in angiogenesis.

Previous reports have suggested that the function and stability of YAP1 can be regulated by methylation, acetylation as well as sumoylation (50). Here we find that stability of YAP1 protein can also be regulated by proline hydroxylation mediated by PHD2, similar to a phenomenon observed in HIF1α. There are three members in the prolyl hydroxylase family namely, PHD1, PHD2, and PHD3 (52, 53). Among them, PHD2 and PHD3 have been shown to be responsive to hypoxia (55, 67) and PHD3 was recently shown to have role in EMT and lung tumor metastasis (68). Our current results confirm that PHD2 is the prolyl hydroxylase that regulates YAP1, as its depletion leads to accumulation of YAP1. While hydroxylation of proline residues increases the stability of proteins by increasing the hydrophilic interactions, it is a vital response mechanism for pathways sensitive to oxygen levels in the vicinity (69). In addition to HIF transcription factors, a recent study showed that additional proteins, like the chromatin modulator *Brd4,* are also regulated by proline hydroxylation (69); YAP1 appears to be a major oncogenic protein that is regulated by the same mechanism. Contrary to a previous publication, depletion or inhibition of PHD2 led to increase in the YAP1 protein stability, as well as its nuclear localization (70).

Our studies further show that YAP1 can interact with VHL tumor suppressor, which is a known E3 ubiquitin ligase that targets HIF1α and HIF2α for proteolytic degradation (13). Among the various tumor types, RCC has frequent mutational loss of the VHL tumor suppressor contributing to poor prognosis (25, 26). Hence, the higher levels of YAP1 in RCC samples suggested a possible correlation between the loss of VHL and the stability of YAP1. It is not clear, though, whether there is a sustained interaction of PHD2 with YAP1 in human lung tumors; while such an interaction is highly possible, it might not contribute significantly to tumor growth, because human lung tumors have high levels of YAP1.

These results therefore indicate a major role of YAP1 in tumor angiogenesis and its contribution toward tumor progression. From the data presented here, it appears that the Hippo kinase-mediated phosphorylation of YAP1 may not be the only major mechanism regulating YAP1 levels in lung adenocarcinomas and that other modifications might regulate YAP1. Under normoxic conditions, YAP1 that escapes proline hydroxylation by PHD2 or phosphorylation by LATS1/2, might translocate into the nucleus to partner with transcription factors such as Oct4 to induce the expression of vital genes like Sox2, which are necessary for the genesis of tumors (Fig. 7E; refs. 27, 71, 72). In contrast, when the tumors become hypoxic, PHD2 has decreased enzymatic activity due to the lack of O_2,_ which might result in increased levels of YAP1; further, as observed in RCCs, mutations in VHL might also lead to the nuclear accumulation of YAP1. It has been found that knockdown of VHL inhibits tumor progression in lung carcinoma (73); it remains to be established if this is mediated by YAP1-dependent mechanisms. Under such circumstances, the increased nuclear YAP1 might promote the expression of multiple genes essential for tumor growth as well as EMT and metastasis, and perhaps additional VEGF family members (Fig. 7E; refs. 27, 46). In summary, the data presented here show that YAP1 is regulated by a novel posttranslational modification that involves proline hydroxylation at a specific region, which is sensitive to oxygen levels. These findings, along with the role of YAP1 in neoangiogenesis, opens new avenues to combat various cancers which are driven by YAP1, as well as tumors like RCC, which show loss of VHL along with elevated YAP1 levels.

## Supplementary Material

Supplementary Methods 1 Details of experimental methods

Supplementary Figure S1 Transient transfection data

Supplementary Figure S2 Single antibody controls for proximity ligation assays

Supplementary Figure S3 Downregulation of VEGF mRNA when YAP1 is depleted

Supplementary Figure S4 Hypoxia-induced reduction in YAP1-PHD2 co-localization

Supplementary Figure S5 High levels of YAP1-HIF1 alpha levels in mouse tumors
