# Supplementary material for: A Novel PHD2/VHL-mediated Regulation of YAP1 Contributes to VEGF Expression and Angiogenesis
Source: Cancer Res Commun. 2022 Jul 12;2(7):624–38. doi: 10.1158/2767-9764.CRC-21-0084 (PMC9351435; doi:10.1158/2767-9764.CRC-21-0084)
Supplement: Supplementary Methods 1 — Details of experimental methods [file crc-21-0084-s01.docx]

**Supplementary Information**

**Supplementary Materials and Methods**

*Antibodies*

Antibodies for MST1 (#14946S), MST2 (#3952S), LATS1(#9153S), LATS2 (#5888S), pYAP1 (S397) (#13619S), PHD2 (#4835S), acetylated histone H3 Lysine 9 (# 9671S) and VHL1 (#68547S) were from Cell Signaling Technologies; mouse monoclonal antibody to actin was from Sigma-Aldrich; E2F1 (sc-193), TEAD2 (sc-67115), TEAD4 (sc-390578), TAZ (sc-48805), VHL1 (sc-135657), 14-3-3 (sc-1657), and Ubiquitin (sc-8017) antibodies were from Santa Cruz Biotechnologies; pYAP1 (S127) (ab76252), Hydroxyproline (ab37067), PHD3 (NB100139SS), and 6XHis tag (ab18184) antibodies were purchased from Abcam. YAP1 antibodies were purchased from Abnova (H00010413-M01) and Abcam (# ab56701). HIF1α antibodies were purchased from Bethyl Laboratories (A300-286A) and BD Pharmingen (610958).

*siRNA and shRNA mediated depletion assays*

The two different siRNAs for YAP1 were purchased from Santa Cruz Biotechnologies (sc-38637) and ThermoFisher Scientific (107951) respectively. siRNAs For TAZ (sc-45232), MST1 (sc-39249), MST2 (sc-39247), LATS2 (sc-37444), HIF1α (sc-35561), PHD2 (sc-45537), PHD3 (sc-45799), TEAD2 (sc-45232) and TEAD4 (sc-96187) were purchased from Santa Cruz Biotechnologies. siRNAs for LATS1 (137593), HIF1α (106498), TAZ (122501), TEAD2 (107179), TEAD4 (115367), PHD2 (133478) and PHD3 (128749) were purchased from ThermoFisher Scientific. Additional siRNAs purchased from Integrated DNA technologies are as follows:

1. LATS1 (199144689)

5’-rGrArArArGrCrUrCrUrGrGrArUrCrUrArUrCrArArArUrAAA-3’

5’-rUrUrUrArUrUrUrGrArUrArGrArUrCrCrArGrArGrCrUrUrUrCrUrU-3’

1. LATS2 (199144692)

5’-rGrGrArCrCrUrUrCrArCrUrGrCrArUrUrArArArArCrArATA-3’

5’ – rUrArUrUrGrUrUrUrUrArArUrGrCrArGrUrGrArArGrGrUrCrCrUrG-3’

1. MST1 (199144695)

5’-rCrArUrGrCrArGrCrUrArGrUrArArArUrGrArUrArGrArATC-3’

5’-rGrArUrUrCrUrArUrCrArUrUrUrArCrUrArGrCrUrGrCrArUrGrArC-3’

1. MST2 (199144698)

5’-rCrCrUrUrGrArArArArUrCrCrArArArGrCrUrArUrUrGrUTG-3’

5’-rCrArArCrArArUrArGrCrUrUrUrGrGrArUrUrUrUrCrArArGrGrUrU-3’

100 pmoles of siRNAs were transfected into cells using Oligofectamine reagent (Invitrogen) as per manufacturer’s protocol. A non-targeting siRNA (AM4611, Ambion) was used as a control for all the transfection experiments. The cells were harvested after 48h post transfection for different assays. All the siRNA experiments were performed thrice.

For the shRNA mediated depletions, the shRNA for VHL1 was obtained from GE Dharmacon (RHS3979-201768476) and viruses were prepared using the psPAX2 and pMD2.G vectors in 293T cells. A549 and H1650 cells were transduced with the supernatant with viral particles and the cells were collected after 72 hours for the indicated experiments.

*RNA Isolation and qRT-PCR analysis*

Total RNA was isolated from the cells by RNeasy miniprep kit from Qiagen following the manufacturer’s protocol. For the sorted cells, RNeasy microprep kit from Qiagen was used. 1 µg of RNA was converted into cDNA using iScript cDNA synthesis kit (Bio-Rad). The changes in the mRNA levels were analyzed using quantitative reverse transcription-PCR (qRT-PCR) that was performed using Bio-Rad CFX96 Real time System. Data was normalized using GAPDH as an internal control and fold change was calculated by 2^-ΔΔCt^ method (1-3). The primers used for the amplification of various genes were as follows:

Flt1 FP 5’-CTGGCTCCTATTAACCCTCCTTA-3’,

Flt1 RP 5’ -ATTTGCCCAGTTTAAGTCTCTCC-3’,

Ang-2 FP 5’-GACTTGAACTTCAGCTCTTGGAA -3’,

Ang-2 RP 5’-TGTAGTTGGATGATGTGCTTGTC -3’,

KDR FP 5’- TGGAAGTGAGTGAAAGAGACACA-3’,

KDR RP 5’- TACTGGTAGGAATCCACAGGAGA -3’,

VEGF FP 5’-GCTCAGAGCGGAGAAAGCAT -3’,

VEGF RP 5’- TTAACTCAAGCTGCCTCGGC-3’,

MMP2 FP 5’-CCGCCTTTAACTGGAGCAAAAA -3’,

MMP2 RP 5’- GATGAGCTTGGGGAAGCCAG-3’,

MMP9 FP 5’-CGGAGCACGGAGACGGGTATC -3’,

MMP9 RP 5’- GGGCAGAGTAGGAGCGGCCCT-3’,

MMP14 FP 5’-GGATACCCAATGCCCATTGGCCAG -3’,

MMP14 RP 5’- CCATTGGGCATCCAGAAGAGAGC-3’,

MMP15 FP 5’-CAGCCCAGCCGCCATATGTC -3’,

MMP15 RP 5’- CTTTCACTCGTACCCCGAAC-3’,

YAP1 FP 5’- CCCAAGACGGCCAACGTGCC-3’,

YAP1 RP 5’- ACTGGCCTGTCGGGAGTGGG-3’,

TAZ FP 5’- TCCCAGCCAAATCTCGTGATG -3’,

TAZ RP 5’- AGCGCATTGGGCATACTCAT -3’,

LATS1 FP 5’- ACCATCCACGGCAAGATAGC -3’,

LATS1 RP 5’- GTGCAGCTCTCCGCTCTAAT -3’,

LATS2 FP 5’- CTAACTGTCGGTGTGGGGAC -3’,

LATS2 RP 5’- CCTCGGGTGCGATGTAGTTT -3’,

MST1 FP 5’- TGGAGACGGTACAGCTGAGG -3’,

MST1 RP 5’- CCTCAGCTGTACCGTCTCCA -3’,

MST2 FP 5’- AGGCTATTTATTGTCCCCTTAGGT-3’

MST2 RP 5’- GACCCTACCTTCACCAAAGCTA-3’,

HIF1α FP 5’- ATCACCCTCTTCGTCGCTTC-3’

HIF1α RP 5’- AGAAGCGGGCGGCAATC-3’,

Oct4 FP 5’ – ACATCAAAGCTCTGCAGAAAGAACT-3’,

Oct4 RP 5’- CTG AAT ACC TTC CCAAAT AGA ACC C-3’,

Nanog FP 5’ – AGAAGGCCTCAGCACCTA-3’,

Nanog RP 5’ – GGCCTGATTGTTCCAGGATT-3’,

GAPDH FP 5’-GGTGGTCTCCTCTGACTTCAACA-3’,

GAPDH RP 5’-GTTGCTGTAGCCAAATTCGTTGT-3’,

*Transfections and luciferase assays*

The A549 and H1650 cells were transiently transfected using FugeneHD (Promega) according to the manufacturer’s protocol. Luciferase assays were carried out 48 h post-transfections using the dual-luciferase assay system (Promega) according to manufacturer’s instructions. Luciferase activity was measured using a luminometer (Glomax MultiJR detection system). For each experiment, the relative luciferase activity was measured as the ratio of the firefly luciferase to *Renilla* luciferase and the fold changes were calculated compared to the control luciferase vector alone from at least three independent experiments. The *VEGFR1 (Flt1), VEGFR2 (KDR), Ang2, MMP2, MMP9, MMP14* and *MMP15* promoter regions were identified and the respective luciferase constructs were used as described in our previous publications(2-4). The 2.6 kb VEGF promoter luciferase (VEGF-luc) construct was a kind gift from Dr. Debabrata Mukhopadhyay^8^. pcDNA4/HisMaxB-YAP1 was a gift from Marius Sudol (Addgene plasmid # 18978)^9^ and HA-HIF1alpha-pcDNA3 was a gift from William Kaelin (Addgene plasmid # 18949)(5).

For mutVEGF-luc construct with HIF1α binding site mutated was generated on the VEGF-luc construct using the QuikChange Lightening multi-site directed mutagenesis kit (Agilent Technologies). The predicted binding site for HIF1α on the VEGF promoter was TACGTG at position -939 to -947 bp upstream of TSS ^11^. The sequence TACGT from -943 to -947 was deleted to prevent binding of HIF1α to VEGF-luc.

*ChIP assays*

ChIP assays were conducted on asynchronous lung adenocarcinoma cell lines as previously described using indicated antibodies(1,2,4,6). The interactions at the promoter were analyzed using qPCR analysis. Each ChIP assay was performed twice. The sequences of the ChIP PCR primers are as follows:

Flt1-ChIP-FP -5’ GCAAATGATCTAGGGCCTCA 3’,

Flt1-ChIP-RP -5’ CGCAGGGCACTTGAACTTTA 3’,

KDR-ChIP-FP -5’ TATCCGCTTCTCCCTTGTGG 3’,

KDR-ChIP-RP -5’ GCGCTGAGCAACTCCAAGATT 3’,

Ang2-ChIP-FP -5’ TCCCTCTGAAAAGGCACACG 3’,

Ang2-ChIP-RP -5’ TTTTTGCCGTGCTAAGCTGG 3’,

MMP2-ChIP-FP -5’CCTCCTAGTAGTACCGCTGC 3’,

MMP2-ChIP-RP -5’ TCACCCCACTTGCCTCTCTC 3’,

MMP9-ChIP-FP 5'-AGATGAAGCAGGGAGAGGAAGC 3',

MMP9-ChIP-RP 5'-CCTCCAGAGGTCAGCCAA 3',

MMP14-ChIP-FP 5'-CCATAGGACTAGCCCAACTATGAG 3',

MMP14-ChIP-RP 5'-GAAGACTGACACCAGATGCTTGC 3',

MMP15-ChIP-FP 5'-GCAGTGGTCTTCAGACACGGAC 3',

MMP15-ChIP-RP 5'-CCAGGCTGGTCTTGAACTCCTG 3',

Myc ChIP FP 5’ CCCCAACAAATGCAATGGGAG 3’,

Myc ChIP RP 5’ CAGAGCGTGGGATGTTAGTG 3’,

VEGF-ChIP-FP 5'-CCAGACTCCACAGTGCATACG 3’,

VEGF-ChIP-RP 5' TTGTGGAGCTGAGAACGGG 3’

*Lysate preparation and IP/Western Blotting*

The asynchronously growing cells were washed twice with ice-cold PBS, scraped and centrifuged at 800 *g* and lysed using M2 lysis buffer (20 mM Tris-HCl pH 6.0, 0.5% NP-40, 250 mM NaCl, 3 mM EGTA and 3 mM EDTA) containing protease inhibitors as described previously (1,6,7)^0^. The protein content was quantitated by Bradford assay (Bio-Rad). Equal amounts of proteins (50 µg) were separated using SDS-PAGE and transferred to nitrocellulose membranes (Bio-Rad Transblot Semi-dry system) and blocked with 5 % nonfat dry milk in PBS with 0.1 % Tween-20 and incubated with the appropriate primary antibodies. 1:3000 diluted HRP-conjugated secondary antibodies (Pierce Biotechnology) were used and signals were detected using ECL (Pierce Biotechnologies).

For co-immunoprecipitation assays, 200 µg of total protein lysate from A549 and H1650 cells were incubated with 4 µg of the indicated antibodies [YAP1 (Abnova), HIF1α (BD Biosciences), PHD2 (Abcam), VHL1 (SCBT)] as described earlier (1,6)^5^. 20% of the protein was loaded as control lysate. The interacting proteins were detected by western blotting.

*Immunofluorescence*

The indicated cells were plated on poly-D-Lysine coated 8-well chamber slides (BD Biosciences) at a density of 5,000 cells/well. For hypoxia experiment, cells were subjected to 1% O_2_ for 24 h. They were then fixed using 10% buffered formalin and permeabilized with 0.2 % Triton-X-100 in PBS at room temperature for 5 min. Immunostaining was performed as described earlier (6,8,9)^3^. For double immunofluorescence, the cells were incubated with primary antibodies for YAP1 (Abcam), HIF1α (Bethyl laboratories) and PHD2 (Abcam). Cells were observed using a Leica TCS SP5 AOBS laser scanning confocal microscope (Leica Microsystems, Germany) and images were acquired at 630X and at 1890X using LAS AF software version 2.6 (Leica Microsystems, Germany). All experiments were repeated three independent times.

*Proximity Ligation Assay*

The asynchronously growing A549 and H1650 cells were plated on poly-D-Lysine coated glass slides at a density of 5000 cells/well (1,9). For the hypoxia treatment, the cells were subjected to 1% O_2_ for 24 h. They are further fixed using 10% buffered formalin and permeabilized with 0.5% Triton-X-100 in PBS for 15 min. The cells were blocked with 5% normal goat serum. The diluted primary antibodies [YAP1 (Abcam), HIF1α (Bethyl laboratories), PHD2 (Abcam) and VHL1 (Cell signaling technologies)] were added to the cells and further incubated at 4^o^C overnight. For the remaining steps, the Duolink assay system was used (Sigma-Aldrich) as described previously^2^.

Proximity ligation assay was conducted on two different tissue microarrays [Lung cancer (IMH-358, Imgenex) Renal cell carcinoma (US BioMax, BC07115a)] as well. For this, the slides were deparaffinized by baking at 62°C for 1 hour and rehydrated as mentioned in our previous publications(1). Antigen retrieval was done by boiling the slide in 0.1 M sodium citrate buffer (pH 6.0) for 25 minutes. The tissue samples were permeabilized with 0.5% Triton-x-100 in PBS for 15 min. The slide was washed with PBS and further blocked with DuoLink blocking buffer provided in the DuoLink assay system (Sigma-Aldrich) for 30 min at 37°C. The incubation with primary antibody followed by secondary antibody and the ligation of the oligonucleotides were performed as described in previous publications. For detection of the signal in tissues, phi29 DNA polymerase (New England Biolabs) was used for amplification at 37°C for 120 min. Also, the tissue samples were treated with anti-Pan-cytokeratin Alexa 488 antibody (e-Biosciences) at 1:50 dilution to detect the tumor areas. The slide was mounted in mounting medium with DAPI (Vectashield). The images were taken at x630 and x1890 with the confocal microscope. The images were analyzed and quantitated using Definiens image analysis software. The fold change in number of spots per cell in normal tissue samples versus tumor samples was compared and graphically represented.

*Coupling of peptide and penetratin*

A peptide corresponding to 10 residues belonging to the Proline hydroxylation region of YAP1 (283-288) was synthesized with a cysteine residue at the C terminus (YAP1 OH-Pro peptide- QPPPLAPQSC) (Genscript). The peptide was incubated with an equal amount of activated penetratin (MP Biologicals) and the conjugate was prepared as per manufacturer’s protocol (1,6,9)^3^. Activated penetratin was also conjugated to a scrambled peptide sequence (Scrambled peptide - QPQPLPASPC) containing the same amino acid content and used as the control peptide for all the assays.

*TMA and Immunohistochemistry*

Immunohistochemistry was conducted on a human renal cancer tissue microarray that was obtained from US BioMax (BC07115a), which had 120 cores covering normal kidney (10 cores) and different grades of renal cell carcinoma (110 cores). Staining was done according to previously published protocols using a primary YAP1 antibody (Abcam) (1,6)^2^; slides were scanned on an Aperio automatic scanning system from Applied Imaging and were scored by a pathologist. The semi-quantitative score was derived by considering both cellularity and intensity of expression (semi-quantitative score = cellularity + intensity). Cellularity was scored as follows: a score of 3 equals to greater than 66% cellularity, a score of 2 equals to 34%–65% cellularity, and a score of 1 equals to less than 33% cellularity. Intensity was scored as follows: a score of 3 equals to strong intensity, a score of 2 equals to moderate intensity, and a score of 1 equals to weak intensity.

*Statistical analysis*

All data have been statistically ana­lyzed using Microsoft Office Excel 2010 (Microsoft Corporation, Redmond, WA) and GraphPad Prism (GraphPad Software, CA). The data presented here is with ± standard error measurements (SEM) values from three independent experiment unless otherwise stated. The statistical comparisons between the groups were carried out by Student’s *t*-test or two-way ANOVA with post-hoc analysis to calculate the *p* value for statistical significance.

**References**

1. Bora-Singhal N, Nguyen J, Schaal C, Perumal D, Singh S, Coppola D*, et al.* YAP1 Regulates OCT4 Activity and SOX2 Expression to Facilitate Self-Renewal and Vascular Mimicry of Stem-Like Cells. Stem cells **2015**;33:1705-18

2. Johnson JL, Pillai S, Pernazza D, Sebti SM, Lawrence NJ, Chellappan SP. Regulation of matrix metalloproteinase genes by E2F transcription factors: Rb-Raf-1 interaction as a novel target for metastatic disease. Cancer research **2012**;72:516-26

3. Schaal C, Pillai S, Chellappan SP. The Rb-E2F transcriptional regulatory pathway in tumor angiogenesis and metastasis. Adv Cancer Res **2014**;121:147-82

4. Pillai S, Kovacs M, Chellappan S. Regulation of vascular endothelial growth factor receptors by Rb and E2F1: role of acetylation. Cancer research **2010**;70:4931-40

5. Kondo K, Klco J, Nakamura E, Lechpammer M, Kaelin WG, Jr. Inhibition of HIF is necessary for tumor suppression by the von Hippel-Lindau protein. Cancer cell **2002**;1:237-46

6. Dasgupta P, Rizwani W, Pillai S, Davis R, Banerjee S, Hug K*, et al.* ARRB1-mediated regulation of E2F target genes in nicotine-induced growth of lung tumors. Journal of the National Cancer Institute **2011**;103:317-33

7. Bora-Singhal N, Perumal D, Nguyen J, Chellappan S. Gli1-Mediated Regulation of Sox2 Facilitates Self-Renewal of Stem-Like Cells and Confers Resistance to EGFR Inhibitors in Non-Small Cell Lung Cancer. Neoplasia **2015**;17:538-51

8. Singh S, Trevino J, Bora-Singhal N, Coppola D, Haura E, Altiok S*, et al.* EGFR/Src/Akt signaling modulates Sox2 expression and self-renewal of stem-like side-population cells in non-small cell lung cancer. Molecular cancer **2012**;11:73

9. Pillai S, Nguyen J, Johnson J, Haura E, Coppola D, Chellappan S. Tank binding kinase 1 is a centrosome-associated kinase necessary for microtubule dynamics and mitosis. Nat Commun **2015**;6:10072
