# Supplementary material for: A Novel PHD2/VHL-mediated Regulation of YAP1 Contributes to VEGF Expression and Angiogenesis
Source: Cancer Res Commun. 2022 Jul 12;2(7):624–38. doi: 10.1158/2767-9764.CRC-21-0084 (PMC9351435; doi:10.1158/2767-9764.CRC-21-0084)
Supplement: Supplementary Figure S1 — Transient transfection data [file crc-21-0084-s02.docx]

**Supplementary Figure 1.**


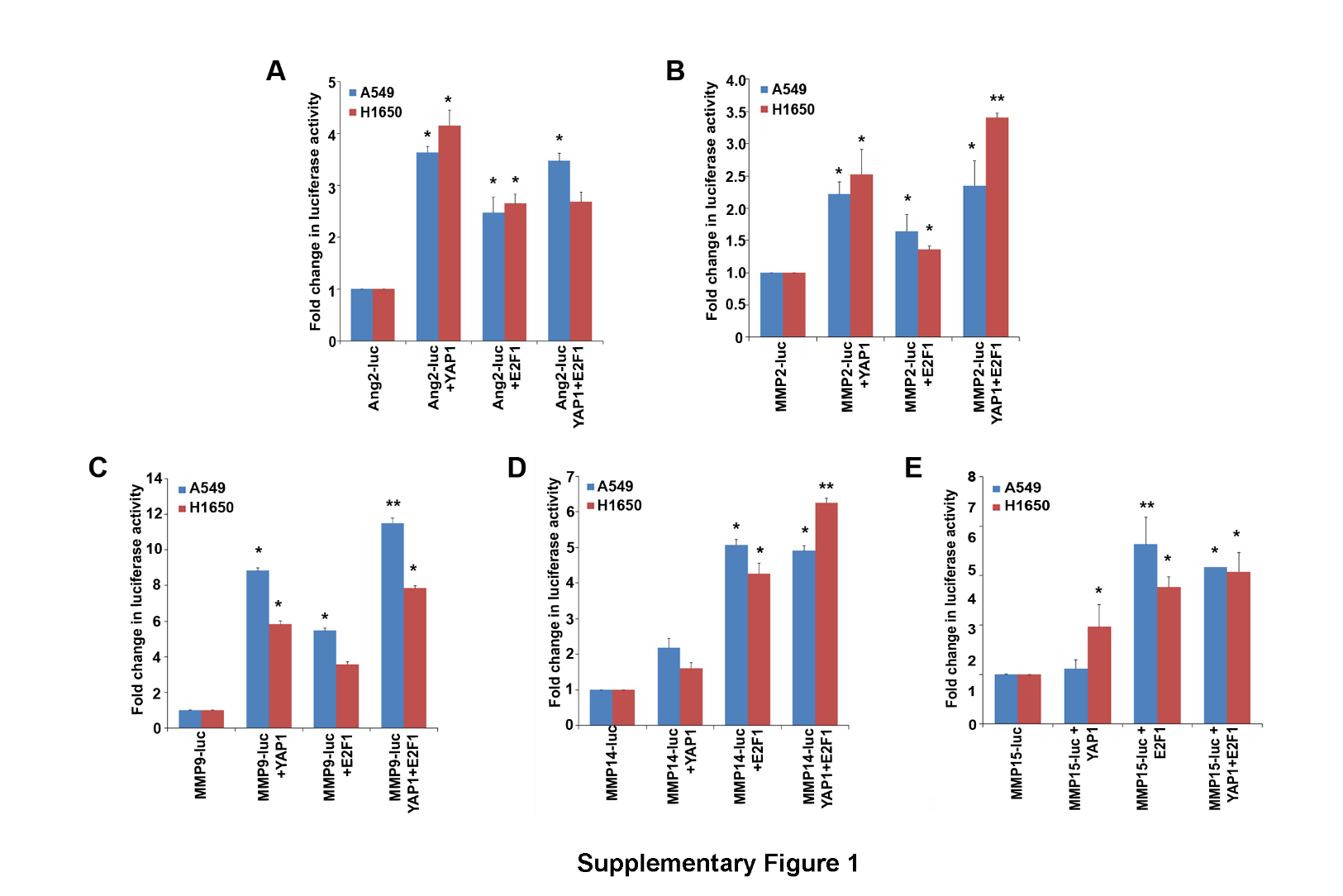


**Supplementary Figure 1:** **(A-E)** Transient transfection of Angiopoietin promoter-luciferase (A), MMP2 promoter-luciferase (B), MMP9 promoter-luciferase (C), MMP14 promoter-luciferase (D) and MMP15 promoter-luciferase constructs (E) with YAP1 and E2F1 over-expression in both A549 and H1650 induced the promoters. The bar graph panels represent mean ± SEM of three independent experiments. * *p* <0.05, ** *p*<0.01 and *** *p*< 0.005 derived by two-way ANOVA with post-hoc test.
