# Supplementary material for: A Novel PHD2/VHL-mediated Regulation of YAP1 Contributes to VEGF Expression and Angiogenesis
Source: Cancer Res Commun. 2022 Jul 12;2(7):624–38. doi: 10.1158/2767-9764.CRC-21-0084 (PMC9351435; doi:10.1158/2767-9764.CRC-21-0084)
Supplement: Supplementary Figure S2 — Single antibody controls for proximity ligation assays [file crc-21-0084-s03.docx]

**Supplementary Figure 2.**


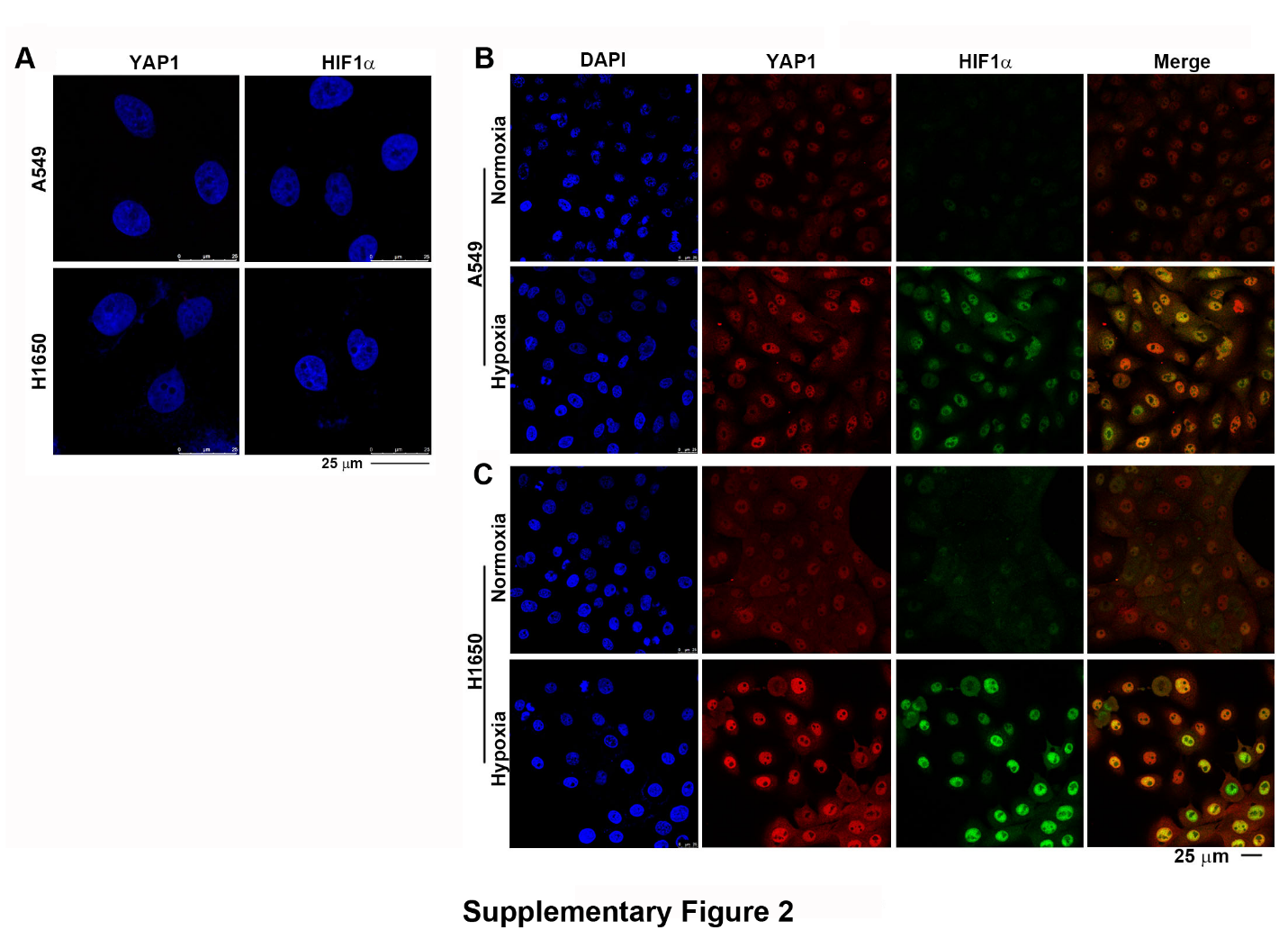


**Supplementary Figure 2: (A)** Single antibody controls for YAP1 and HIF1α for the PLA experiment performed on A549 and H1650 cells presented in Figure 6A. **(B and C)** Double immunofluorescence assays revealed the co-localization of YAP1 with HIF1α that is increased when A549 (B) and H1650 (C) cells were exposed to hypoxia (1% O_2_) for 24h. Scale bar 25 μm.
