# Supplementary material for: A Novel PHD2/VHL-mediated Regulation of YAP1 Contributes to VEGF Expression and Angiogenesis
Source: Cancer Res Commun. 2022 Jul 12;2(7):624–38. doi: 10.1158/2767-9764.CRC-21-0084 (PMC9351435; doi:10.1158/2767-9764.CRC-21-0084)
Supplement: Supplementary Figure S3 — Downregulation of VEGF mRNA when YAP1 is depleted [file crc-21-0084-s04.docx]

**Supplementary Figure 3.**


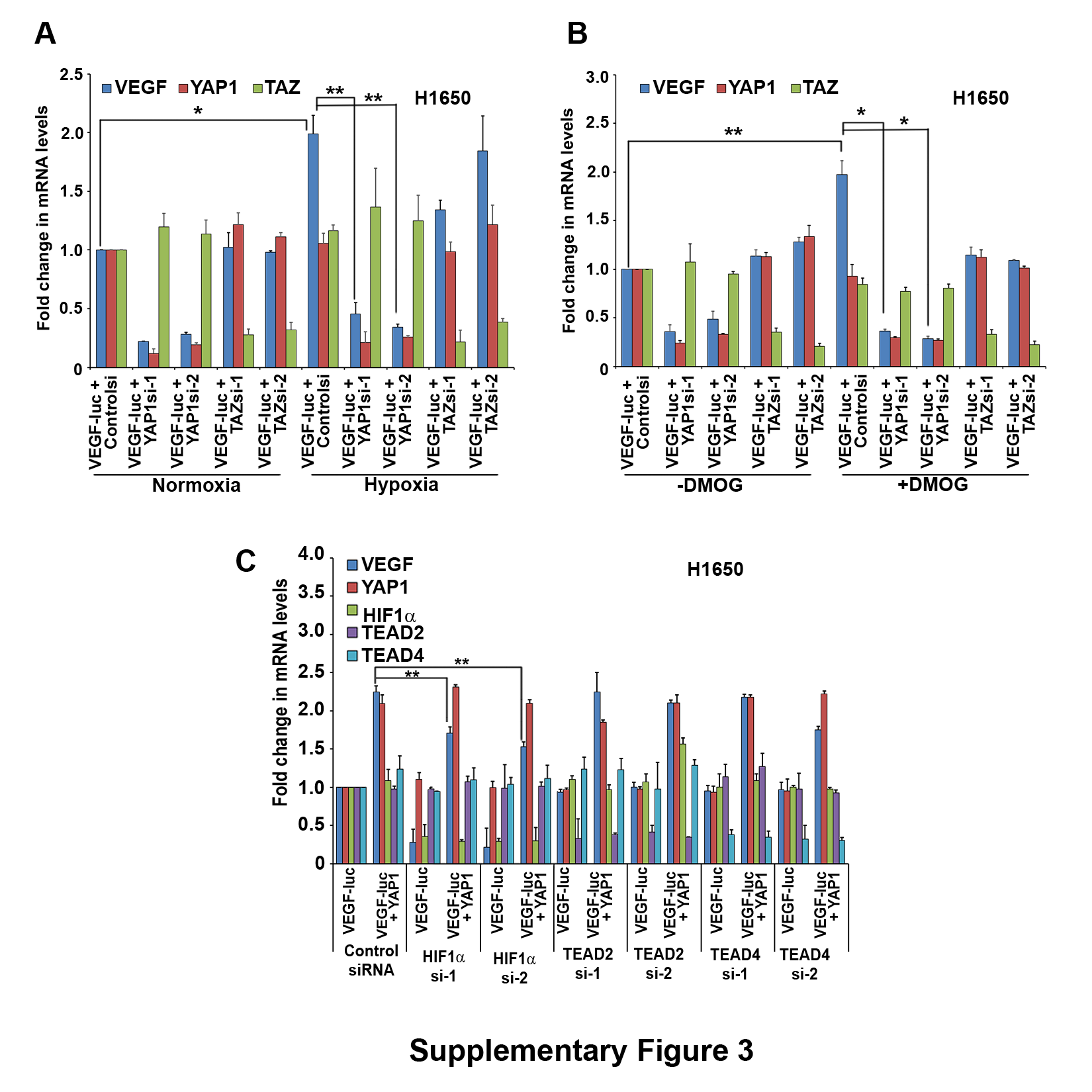


**Supplementary Figure 3:** **(A)** qRT-PCRs showed decreased expression of endogenous *VEGF* mRNA in H1650 cells subjected to hypoxia when transfected with *YAP1* siRNA. No change was observed when *TAZ* was depleted in H1650 cells. **(B)** Similarly, qRT-PCR analysis showed that the expression of endogenous *VEGF* mRNA was reduced when H1650 cells were treated with *YAP1* siRNA but not *TAZ* siRNA even in the presence of DMOG. **(C)** Reduced expression of *VEGF* mRNA was observed with YAP1 overexpression when *HIF1α* was depleted but no change was observed in *TEAD2* or *TEAD4* depletion. The bar graph panels represented mean ± SEM of three independent experiments. * *p* <0.05 and ** *p*<0.01 derived by two-way ANOVA with post-hoc test.
