# Supplementary material for: A Novel PHD2/VHL-mediated Regulation of YAP1 Contributes to VEGF Expression and Angiogenesis
Source: Cancer Res Commun. 2022 Jul 12;2(7):624–38. doi: 10.1158/2767-9764.CRC-21-0084 (PMC9351435; doi:10.1158/2767-9764.CRC-21-0084)
Supplement: Supplementary Figure S4 — Hypoxia-induced reduction in YAP1-PHD2 co-localization [file crc-21-0084-s05.docx]

**Supplementary Figure 4.**


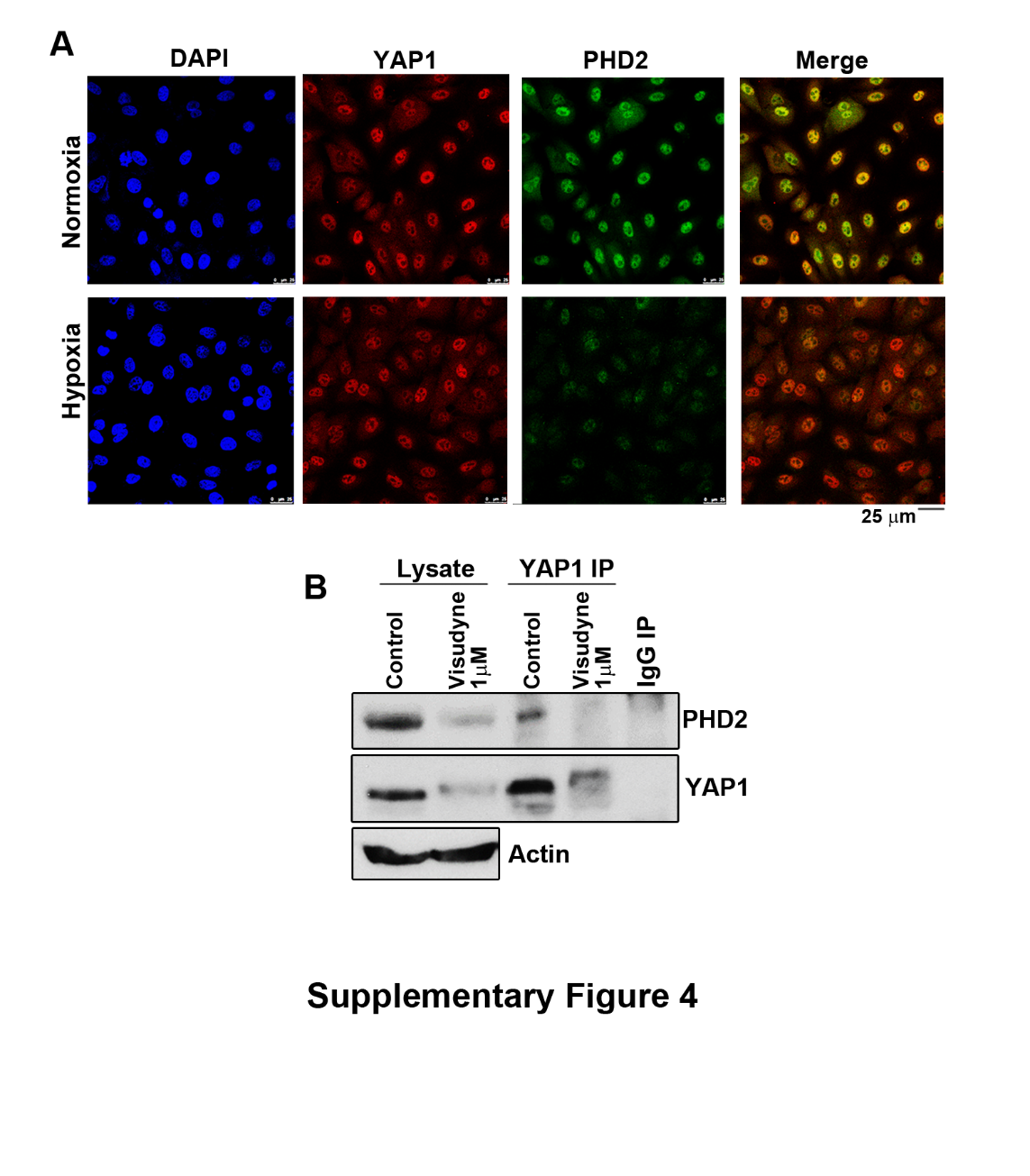


**Supplementary Figure 4: (A)** Higher YAP1 and PHD2 co-localization observed during normoxia that was reduced when A549 cells were treated with hypoxia (1% O_2_). Scale bar 25 μm. **(B)** Immunoprecipitation assay with YAP1 antibody in 1 μM visudyne treated A549 cells showed a reduced interaction of YAP1 with PHD2 in visudyne treated cell lysate as compared to the control cell lysate. 1 μM Visudyne treatment was carried out for 24h in A549 cells.
