# Supplementary material for: A Novel PHD2/VHL-mediated Regulation of YAP1 Contributes to VEGF Expression and Angiogenesis
Source: Cancer Res Commun. 2022 Jul 12;2(7):624–38. doi: 10.1158/2767-9764.CRC-21-0084 (PMC9351435; doi:10.1158/2767-9764.CRC-21-0084)
Supplement: Supplementary Figure S5 — High levels of YAP1-HIF1 alpha levels in mouse tumors [file crc-21-0084-s06.docx]

**Supplementary Figure 5.**


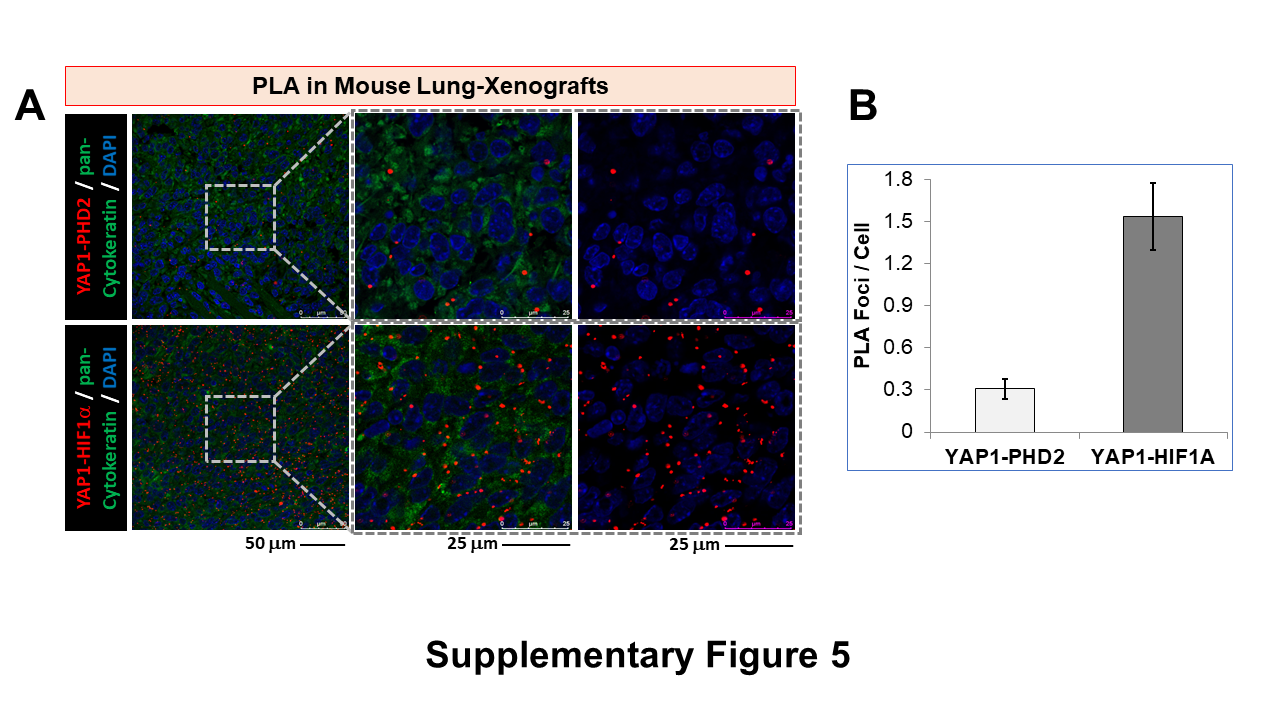


**Supplementary Figure 5: (A)** PLA studies in mouse lung-xenograft tissues show that YAP1-HIF1α interaction is relatively stronger and abundant (lower panel) as it shows more PLA-foci in comparison to YAP1-PHD2 interaction (upper panel). These experiments indeed demonstrate that YAP1 functional network is highly active in association with HIF1α under hypoxic conditions, *in vivo* within the actual tumor core. **(B)** Quantitation of PLA foci per cell for the above-stated interactions in the tissue sections, mean ± SD of three independent PLA experiments. Control lung-xenografted tissue sections were obtained from three C57BL/6 mice, around 200 cells were considered for pursuing the statistics for each stated interaction per section.
